# Evidence and Gap Map of Whole‐School Interventions Promoting Mental Health and Preventing Risk Behaviours in Adolescence: Programme Component Mapping Within the Health‐Promoting Schools Framework: An evidence and gap map

**DOI:** 10.1002/cl2.70024

**Published:** 2025-03-10

**Authors:** Roshini Balasooriya Lekamge, Ria Jain, Jenny Sheen, Pravik Solanki, Yida Zhou, Lorena Romero, Margaret M. Barry, Leo Chen, Md Nazmul Karim, Dragan Ilic

**Affiliations:** ^1^ School of Public Health and Preventive Medicine Monash University Melbourne Victoria Australia; ^2^ Alfred Mental and Addiction Health, Alfred Health Melbourne Victoria Australia; ^3^ St Vincent's Hospital Melbourne Victoria Australia; ^4^ Eastern Health Melbourne Victoria Australia; ^5^ Department of Psychiatry School of Translational Medicine Monash University Melbourne Victoria Australia; ^6^ Ian Potter Library, Alfred Health Melbourne Victoria Australia; ^7^ Health Promotion Research Centre University of Galway Galway Ireland

**Keywords:** adolescence, health‐promoting schools framework, mental disorders, mental health, risk behaviours, whole‐school approach

## Abstract

Adolescence is a vulnerable period for the onset of mental disorders and risk behaviours. Whole‐school interventions hold vast potential in improving mental health and preventing risk behaviours in this developmentally‐sensitive cohort. Modelled on the World Health Organisation's Health‐Promoting Schools Framework, whole‐school interventions aspire for change across eight domains: (i) school curriculum, (ii) school social‐emotional environment, (iii) school physical environment, (iv) school governance and leadership, (v) school policies and resources, (vi) school and community partnerships, (vii) school health services and (viii) government policies and resources. Through embodying a systems‐based approach and involving the key stakeholders in an adolescent's life, including their peers, parents and teachers, whole‐school interventions are theoretically more likely than other forms of school‐based approaches to improve adolescent mental health and prevent risk behaviours. However, vague operationalisation of what is to be implemented, how and by whom presents challenges for stakeholders in identifying concrete actions for the eight domains and thus in realising the potential of the Framework. Mapping how whole‐school interventions operationalise the eight domains enables appraisal of current practice against the recommendations of the Health‐Promoting Schools Framework. This facilitates identification of critical evidence gaps in need of research, with the aim of fostering optimal translation of the Framework into practice to promote mental health and prevent risk behaviours in adolescence. Our EGM's objective was to map how randomised controlled trials of whole‐school interventions promoting mental health and preventing risk behaviours in adolescence addressed the eight domains of a whole‐school approach. Our EGM was conducted in accordance with a pre‐registered protocol (PROSPERO ID: CRD42023491619). Eight scientific databases were searched: Ovid MEDLINE, Ovid Embase, Ovid PsycINFO, Ovid Emcare, CINAHL, ERIC, CENTRAL and Scopus. Expert‐recommended sources of the grey literature were also searched, including the Blueprints for Healthy Youth Development registry of evidence‐based positive youth development programmes and the SAMHSA Evidence‐Based Practice Resource Centre. To be included in our EGM, studies had to involve randomised controlled trials or cluster randomised controlled trials comprising students aged 12 to 18. Interventions had to demonstrate a whole‐school approach promoting mental health and/or preventing risk behaviours, including at least one program component addressing each of the curriculum‐, ethos and environment‐, and community‐levels of a whole‐school approach. Studies had to include an active or inactive comparator. Studies had to report on at least one of the mental health and/or risk behaviour outcomes detailed in the WHO‐UNICEF Helping Adolescents Thrive Initiative, which includes positive mental health, mental disorders, mental health literacy, substance use, bullying and aggression. Two independent reviewers screened search results, with disagreements resolved by a third reviewer on the research team. Risk‐of‐bias assessments were completed by two independent reviewers for each included study using the Cochrane risk‐of‐bias tool, with disagreements resolved by a third reviewer on the research team. Data extraction for each included study was completed independently by two reviewers, in accordance with a prespecified template. Data extraction included study characteristics and intervention components, the latter of which was mapped against the eight domains of a whole‐school approach. 12, 897 records were identified from the searches. A total of 28 studies reported in 58 publications fulfilled the inclusion criteria. The majority of interventions implemented by studies classified as either substance use prevention (10 of 28 studies) or multiple risk behaviour interventions (8 of 28 studies). The majority of studies involved students in lower secondary school grade levels, with only 5 of 28 studies targeting students in grades 10 to 12. The majority of studies were set in high‐income countries, with minimal representation of low‐ and middle‐income countries (5 of 28 studies). The interventions implemented by studies ranged from 9 weeks to 3 years in duration. Though 100% of studies involved students in the evaluation stage and 61% in the implementation of intervention strategies, only 39% involved students in the planning and 29% in the design of whole‐school interventions. Significant variability existed in how frequently whole‐school interventions addressed each of the eight domains, ranging from 7% to 100%. This included 100% of interventions implemented by studies addressing the school curriculum domain, 64% the school social‐emotional environment domain, 46% the school physical environment domain, 50% the school governance and leadership domain, 61% the school policies and resources domain, 93% the school and community partnerships domain, 29% the school health services domain and 7% the government policies and resources domain. Despite different intervention foci, there was a clear overlap in whole‐school intervention strategies within each domain. Our EGM identifies several critical foci for future research. These include the need to investigate (i) whether certain domains of a whole‐school approach are critical to intervention success; (ii) whether addressing more domains translates to greater impact; and (iii) the relative effectiveness of common intervention strategies within each domain to enable the most effective to be prioritised. Our EGM identifies the need for greater investment in older adolescent populations and those from low‐ and middle‐income countries. Finally, we encourage stakeholders including researchers, schools, public health and policy makers to consider four crucial factors in the design and planning of whole‐school interventions and to investigate their potential impact on intervention success. These include: (i) the provision of training and support mechanisms for those implementing interventions; (ii) the decision between single‐issue versus multiple‐issue prevention programs; (iii) the optimal intervention duration; and (iv) the involvement of adolescents in the design and planning of whole‐school interventions to ensure that interventions reflect their real‐world needs, preferences and interests.

## 1 Plain Language Summary

Declarative title: Evidence and gap map finds significant variability with which randomised trials on whole‐school approaches promoting mental health and preventing risk behaviours in adolescence integrate all 8 dimensions of a whole‐school approach.

### 1.1 EGM in Brief

There is significant variability with which randomised controlled trials of whole‐school interventions addressed the eight domains of a whole‐school approach, ranging from 7% of interventions explicitly referencing the government policies and resources domain to 100% addressing the school curriculum domain. Despite different intervention foci, there is a clear overlap of intervention strategies within each domain.

### 1.2 What Is This EGM About?

Adolescence is a vulnerable period for the onset of mental disorders and risk behaviours. Whole‐school interventions hold vast potential in improving mental health and preventing risk behaviours in this vulnerable period. These interventions are modelled on the World Health Organisation's Health‐Promoting Schools Framework, which advocates for change across eight domains. These include the (i) school curriculum, (ii) school social‐emotional environment, (iii) school physical environment, (iv) school governance and leadership, (v) school policies and resources, (vi) school and community partnerships, (vii) school health services, and (viii) government policies and resources. Through offering a holistic approach to health promotion and prevention and involving the key stakeholders in an adolescent's life, including their peers, parents and teachers, whole‐school interventions are theoretically more likely than other forms of school‐based approaches to improve mental health and prevent risk behaviours in adolescents. However, vague instruction on what is to be implemented, how and by whom presents challenges for stakeholders in identifying concrete intervention strategies to address the eight domains and thus in realising the potential of the Framework. Mapping how whole‐school interventions address the eight domains enables appraisal of current practice against the recommendations of the Health‐Promoting Schools Framework. This allows for identification of critical evidence gaps in need of future research, with the aim of fostering optimal translation of the Framework into practice to promote mental health and prevent risk behaviours in adolescence.

### 1.3 What is the Aim of This EGM?

To map how randomised controlled trials of whole‐school interventions promoting mental health and preventing risk behaviours in adolescence address the eight domains of a whole‐school approach.

### 1.4 What Studies Are Included?

A total of 28 studies reported in 58 publications were included in our EGM. The majority of interventions implemented by studies classified as either substance use prevention or multiple risk behaviour interventions. Most studies targeted students in lower secondary school grade levels, with only 5 studies targeting students in grades 10–12. There was minimal representation of studies set in low‐ and middle‐income countries (5 of 28 studies). The interventions implemented by studies ranged from 9 weeks to 3 years in duration.

### 1.5 What Are the Main Findings?

Though 100% of studies involved students in the evaluation stage and 61% in the implementation of intervention strategies, only 39% involved students in the planning and 29% in the design of whole‐school interventions. There was significant variation in how frequently whole‐school interventions addressed each of the eight domains, ranging from 7% to 100%. This included 100% of interventions addressing the school curriculum domain, 64% the school social‐emotional environment domain, 46% the school physical environment domain, 50% the school governance and leadership domain, 61% the school policies and resources domain, 93% the school and community partnerships domain, 29% the school health services domain and 7% the government policies and resources domain. Despite different intervention foci, there was a clear overlap in the intervention strategies implemented within each domain.

### 1.6 What Do the Findings of the Map Mean?

Our EGM identifies several critical gaps for future research. These include the need to investigate (i) whether certain domains of a whole‐school approach are critical to intervention success; (ii) whether addressing more domains translates to greater impact; and (iii) the relative effectiveness of overlapping intervention strategies within each domain, so that the most effective can be prioritised. Our EGM identifies the need for more studies to involve older adolescent populations and those from low‐ and middle‐income settings. We highlight four crucial factors for stakeholders to consider in the design and planning of whole‐school interventions, alongside exploration of their potential impact on intervention success. These include (i) the provision of training and support mechanisms for those delivering interventions; (ii) the decision of whether an intervention targets a single issue or multiple issues; (iii) the optimal time duration for implementation; and (iv) the involvement of students in the design and planning phases to ensure that interventions mirror their real‐world needs, preferences and interests.

### 1.7 How Up‐to‐Date Is This EGM?

The searches were all conducted on the 4th of September 2023.

## 2 Background

### 2.1 The Problem, Condition or Issue

Adolescence is a period of rapid development, where individuals experience profound psychological, social and physical change (Dray et al. 2017). This developmental period has been associated with a heightened susceptibility to poor mental health and risk behaviour outcomes (Solmi et al. 2022; WHO and UNICEF 2021). Global experts have outlined an agenda of critical outcomes to consider in adolescence, with mental health outcomes comprising positive mental health, mental disorders and mental health literacy; and risk behaviours outcomes comprising bullying, aggression and substance use (WHO and UNICEF 2021; Skeen et al. 2019). This is due to the high‐prevalence of mental disorders (Solmi et al. 2022), bullying (Modecki et al. 2014), aggression (Modecki et al. 2014) and substance use (OECD and European Union 2022; OECD and European Union 2024), and low‐prevalence of positive mental health (Keyes 2006) and mental health literacy (Lam 2014) among this developmentally sensitive cohort.

The high rates of poor mental health and risk behaviour outcomes in adolescence necessitates effective promotive and preventive strategies to mitigate these negative outcomes. While mental health and risk behaviours represent two distinct constructs, it is imperative to acknowledge that the two are highly inter‐related. For example, studies have highlighted the bi‐directional relationship between mental health and bullying, with adolescent victims of bullying significantly more likely to experience poor mental health (Owusu et al. 2011), while adolescents with mental disorders are more likely to be victims of bullying (Maïano et al. 2016). Scholars argue that the literature's hyper‐focus on single‐issue interventions and outcomes fails to recognise this inter‐connectivity as well as the substantial overlap in the risk and protective factor profile for mental health and risk behaviour outcomes in adolescence (Barry et al. 2017; Skeen et al. 2019). Single‐issue interventions have additionally been construed as impractical in real‐life settings, perpetuating the stress related to competing public health priorities among stakeholders and serving as more likely to be ‘crowded out’ by other interventions as policy and funding priorities change (Boustani et al. 2015; Skeen et al. 2019). Global experts have thus emphasised the need for a holistic approach to promoting mental health and preventing risk behaviours in adolescence (WHO and UNICEF 2021; Skeen et al. 2019).

**Figure 1:**
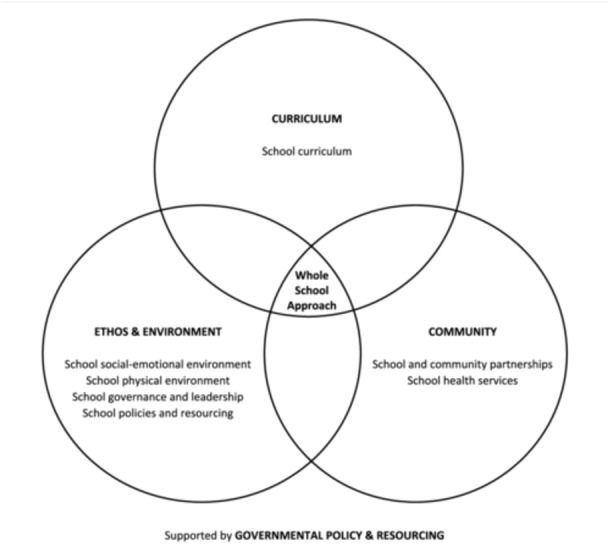
Global standards of the health promoting schools framework (reproduced from Balasooriya Lekamge et al. (2025) as published in Springer Nature, under the CC BY license: https://creativecommons.org/licenses/).

Whole‐school interventions are well‐positioned to realise this aim, representing a departure from a reductionist focus on single issues, risk factors and linear causality, towards a more holistic understanding of health and wellbeing (Samdal and Rowling 2012). Modelled on the World Health Organisation's Health‐Promoting Schools Framework, whole‐school interventions champion ‘an approach which goes beyond the learning and teaching in the classroom to pervade all aspects of the life of a school’ (WHO & UNESCO 2021b). The Health‐Promoting Schools Framework is inspired by the socio‐ecological model, which acknowledges that health and wellbeing are determined by a complex interplay of individual, interpersonal, community and societal factors and therefore advocates for a holistic approach to health promotion and prevention (McLeroy et al. 1988; Samdal and Rowling 2012). In line with this, the Health‐Promoting Schools Framework campaigns for change across eight domains: (i) school curriculum, (ii) school social‐emotional environment, (iii) school physical environment, (iv) school governance and leadership, (v) school policies and resources, (vi) school and community partnerships, (vii) school health services, and (viii) government policies and resources (WHO & UNESCO 2021b). The eight domains can be re‐organised into four levels (Figure 1), with an intervention qualifying as ‘whole‐school’ provided that it includes at least one programme component addressing each of the curriculum‐, ethos and environment‐, and community‐levels (Goldberg et al. 2019; Langford et al. 2015). Through championing a systems‐based approach and involving the prominent stakeholders in an adolescent's life, including their peers, parents and teachers, scholars advocate that whole‐school interventions are theoretically more likely than other forms of school‐based interventions to promote mental health and prevent risk behaviours among school‐aged populations (Barry et al. 2019; Goldberg et al. 2019; Samdal and Rowling 2012).

### 2.2 Why It Is Important to Develop This EGM?

Despite the theoretical promise of whole‐school interventions in promoting mental health and preventing risk behaviours in adolescence, scholars argue that the term ‘whole school’ and its constituent eight domains have not been adequately disambiguated (O'Reilly et al. 2018; Wigelsworth et al. 2022). General guidelines that describe the rationale, objectives and indicators (Table 1) for each of the domains exist (WHO & UNESCO 2021b); however, vague operationalisation of what is to be implemented, how it should be implemented and by whom, presents challenges for stakeholders in identifying concrete actions for each domain (Samdal and Rowling 2012). For example, a workshop with teachers implementing a whole‐school intervention identified that, despite the curriculum domain being favourably received and widely used, their greatest challenge was in addressing the remaining domains of a whole‐school approach (Wyn et al. 2000). This ambiguity has led to significant variation in the operationalisation of the domains across schools (Samdal and Rowling 2012), with some whole‐school interventions demonstrating only passive efforts with relation to certain domains (Langford et al. 2015). Given the endorsement of whole‐school interventions across multiple global contexts (WHO & UNSECO 2021a), it is critical to foster an understanding of how whole‐school interventions promoting mental health and preventing risk behaviours in adolescence have been operationalised in practice. This will enable appraisal of current practice against the recommendations of the Health‐Promoting Schools Framework, highlighting areas in need of improvement to facilitate optimal translation of the Framework into practice to promote mental health and prevent risk behaviours in this developmentally‐sensitive cohort.

**Table 1:** Overview of the eight domains of the health‐promoting schools framework, adapted from the World Health Organisation and the United Nations Educational, Scientific and Cultural Organisation (WHO and UNESCO 2021b).

**SCHOOL CURRICULUM (** ***Curriculum‐Level)***
The school curriculum supports students' physical, psychological and social wellbeing. This is achieved through both *explicit education* (improving students' health knowledge, skills, attitudes and behaviours) and *implicit education* (practice of an inclusive and participatory pedagogy). Examples of explicit education include social‐emotional skills, healthy relationship skills, mental health literacy, substance use and violence prevention. The school curriculum is age‐appropriate, inclusive, gender‐responsive, evidence‐informed, rights‐based and free from personal biases. Staff receive training and support to improve their health literacy, meet students' physical, psychological and social needs, know referral options and use appropriate learning and teaching strategies. Both implicit and explicit education are regularly planned, monitored for progress and performance, and revised where necessary.
**SCHOOL SOCIAL‐EMOTIONAL ENVIRONMENT (** ***Ethos & Environment‐Level)***
The school's social‐emotional environment is safe, supportive and inclusive, with this ethos embodied by all members of the school community in their interactions. Students feel respected within and connected to the school community. All stakeholders in the school and local community are involved and reach consensus on the desired elements of the social‐emotional environment. School policies provide clear directions for the desired school climate, with no tolerance of bullying and discrimination, harassment or corporal punishment. Professional training is provided to teachers to develop the skills necessary in cultivating the desired school climate. Mechanisms exist to identify and respond to any disruption in the school ethos and the ethos is regularly monitored and revised.
**SCHOOL PHYSICAL ENVIRONMENT (** ***Ethos & Environment‐Level)***
The school physical environment is safe, secure, inclusive, healthy and accessible for all students and the school community before, during and after school hours. These standards are embedded in school policies and consistent with national regulations. The school physical environment includes (i) school grounds, facilities and equipment (e.g. classrooms, cafeteria, sports facilities), (ii) transport facilities (e.g. footpaths, carparks, school buses), (iii) community facilities used by the school (e.g. sports fields, gardens, swimming pools), and (iv) local shops and businesses used by the school community (e.g. corner shops). Both *direct influences* (e.g. compliance with health and safety regulations) and *indirect influences* (e.g. advertisements, shops selling alcohol and other drugs) are considered. Examples of initiatives include the presence of spaces for play, spaces for spiritual practice, quiet spaces and buddy benches. There is adequate investment by way of resources, training and funding to maintain the desired physical and virtual environments, monitor compliance with required standards and regulations, and initiate corrective actions where necessary.
**SCHOOL GOVERNANCE AND LEADERSHIP (** ***Ethos & Environment‐Level)***
The school board, staff, students, parents and caregivers are involved in a collaborative model of school leadership, with the leadership team provided with training and resources to support a health‐promoting school. Training includes in the range of social determinants, health risk and protective factors, health problems that affect students, student resilience, diversity and inclusion, and the use of monitoring and evaluation systems. The team meets regularly to review and integrate the needs, preferences and interests of the school community into school operations. The team's progress and performance are monitored, and revised where needed.
**SCHOOL POLICIES AND RESOURCING (** ***Ethos & Environment‐Level)***
School policies are equitable, inclusive, rights‐based and informed by evidence and a local needs assessment. School policies, plans and resource allocation are consistent with a school's commitment to being a health‐promoting school. These ensure structure and clear communication for the school community, as well as monitoring and evaluation to confirm that policies are effective and sustainable.
**SCHOOL AND COMMUNITY PARTNERSHIPS (** ***Community‐Level*** **)**
The school collaborates with the local community to achieve a health‐promoting school. This includes collaboration and consultation (i) within the school community (e.g. between staff and parents and caregivers) and (ii) between the school and local community (e.g. between students, staff, NGOs, local businesses and government). The school invests in building students' capacity to execute health‐promoting activities and serve as advocates and agents of change in the school and local community. Health‐promoting partnerships are planned and monitored for progress and performance by the school leadership team, with revisions made where necessary.
**SCHOOL HEALTH SERVICES (** ***Community‐Level*** **)**
School‐based or school‐linked health services exist that meet the physical, psychological, social and educational needs of all students. Examples of such services include health promotion, education and preventive interventions, and clinical assessment and management of health conditions in domains such as mental health, sexual and reproductive health. Services are adequately resourced, timely, safe, age‐appropriate, gender‐responsive, culturally sensitive, evidence‐based and rights‐based. This is reflected in school policies and is consistent with national regulations. Investment in school health services is evidenced in resource allocation, training and funding, with explicit agreement between the education and health sectors around roles, responsibilities, referral pathways and sources of funding. A system exists for planning services and monitoring their progress, performance, quality assurance and compliance with regulations.
**GOVERNMENT POLICY AND RESOURCING (** ***Governmental‐Level)***
Investment in making every school a health‐promoting school is reflected at national, subnational and local levels of government. This includes a clear policy commitment, adequate resource allocation, ensuring intersectoral collaboration between the education and health sectors, accountability for the implementation of health‐promoting schools and a system for monitoring and evaluation.

## 3 Objective

The objective of our EGM is to map how whole‐school interventions promoting mental health and preventing risk behaviours in adolescence address the eight domains of a whole‐school approach. While a 2015 systematic review of whole‐school interventions promoting health and wellbeing in school‐aged youth provided a brief overview of how whole‐school interventions address the curriculum, ethos and environment, and community macro‐levels of a whole‐school approach (Langford et al. 2015), to the best of our knowledge, this is the first attempt at a comprehensive synthesis of intervention strategies according to the eight domains of a whole‐school approach.

## 4 Methods

### 4.1 Evidence and Gap Map: Definition and Purpose

EGMs offer a systematic process by which to identify and map existing literature on a broad topic. This fosters understanding of current practice and identification of critical gaps in the evidence‐base (Grant and Booth 2009). This knowledge benefits a wide range of stakeholders including researchers, public health and policy makers in making informed decisions about primary and secondary research foci, as well as priorities for policy and practice.

### 4.2 Framework Development and Scope

This systematic EGM was conducted in accordance with a pre‐registered protocol (PROSPERO ID: CRD42023491619). Given that whole‐school interventions are modelled on the Health‐Promoting Schools Framework, this Framework served as the basis of this EGM. The eight domains of the Health‐Promoting Schools Framework and related seminal documents (WHO & UNESCO 2021b; Samdal and Rowling 2012; Barry et al. 2019) guided the development of the data extraction template, as well as the coding of data to each domain. The recommendations of the Framework further served as an appraisal tool to enable identification of evidence gaps. The EGM includes an interactive visual map, where rows separate studies by the intervention type they implement, and columns separate studies by which domains of a whole‐school approach they address. Users can apply filters to view studies based on the target population, geographical location, duration of interventions, and the availability of training and support mechanisms for those implementing interventions. The EGM is a valuable tool for stakeholders including schools, researchers, public health and policy makers. It allows easy navigation of the evidence‐base, helping stakeholders identify relevant studies on whole‐school interventions that promote mental health and prevent risk behaviours in adolescence, in addition to the critical gaps that exist in the evidence‐base.

### 4.3 Dimensions

The inclusion criteria for our review is detailed in Table 2.

**Table 2:** Inclusion criteria mapped by the PICO framework.

PICO category	Inclusion criteria
Population	Studies comprising students aged 12 to 18 were eligible for inclusion in the review. Where grade level was used in lieu of age, studies that recruited students in grades 7 to 12 were included in the review provided that the mean age of the participant population was within 12 to 18 years.
Intervention	Interventions had to demonstrate a whole‐school approach promoting mental health and/or preventing risk behaviours, including at least one programme component addressing each of the curriculum‐, ethos and environment‐, and community‐levels of a whole‐school approach (Goldberg et al. 2019; Langford et al. 2015). Similar to previous reviews (Langford et al. 2015), whole‐school interventions had to be universal in nature, including all students within a particular classroom or grade level, rather than a select high‐risk group.
Comparison	Studies had to include an active or an inactive comparator.
Outcome	Studies had to report on at least one of the mental health and/or risk behaviour outcomes detailed in the WHO‐UNICEF Helping Adolescents Thrive Initiative (WHO and UNICEF 2021; Skeen et al. 2019), including (i) positive mental health: outcomes related to emotional, psychological and social wellbeing (Keyes 2013); (ii) mental disorders: outcomes related to mental disorders, such as anxiety and depression; (iii) substance use: outcomes related to smoking, alcohol and recreational drug use; (iv) bullying: outcomes related to being a victim of in‐person or online bullying; (v) aggression: outcomes related to being a perpetrator of in‐person or online aggression; (vi) mental health literacy: outcomes related to the understanding of how to achieve and sustain positive mental health, the understanding of mental disorders and available treatments, the stigma surrounding mental disorders and the ability to seek help for mental disorders (Wei et al. 2015).
Trial design	Randomised controlled trials.

### 4.4 Search Methods and Sources

The Campbell Searching for Studies Guide was consulted in the development of our search strategy (Kugley et al. 2017). Searches were conducted by an experienced research librarian across the following databases: Ovid MEDLINE, Ovid Embase, Ovid PsycINFO, Ovid Emcare, CINAHL, ERIC, CENTRAL and Scopus. Grey literature searches were also conducted on online platforms recommended by experts in the subject area (Barry et al. 2019), including the SAMHSA Evidence‐Based Practice Resource Centre (https://www.samhsa.gov/resource-search/ebp) and Blueprints for Healthy Youth Development registry of evidence‐based positive youth development programmes (http://www.blueprintsprograms.com/programs). Both database‐specific subject headings and free text terms were used as part of the search strategy, canvassing the following concept areas: (i) Whole‐School Approaches; (ii) Mental Health and Risk Behaviours; and (ii) Adolescent and/or Secondary School Age populations. Forwards and backwards citation tracking were additionally completed. Studies were restricted to include peer‐reviewed articles published in English, with the exclusion of editorials, letters, comments, newspaper articles, reviews and case reports. There were no restrictions placed on the date of publication. Searches were adapted as appropriate to the specifications of each of the eight databases. The final searches were undertaken from database inception until 4th of September, 2023. The list of included studies from related systematic reviews were checked to identify additional studies. The search strategy for each database is provided in Supporting Information S4.

### 4.5 Data Collection and Analysis

**Figure 2:**
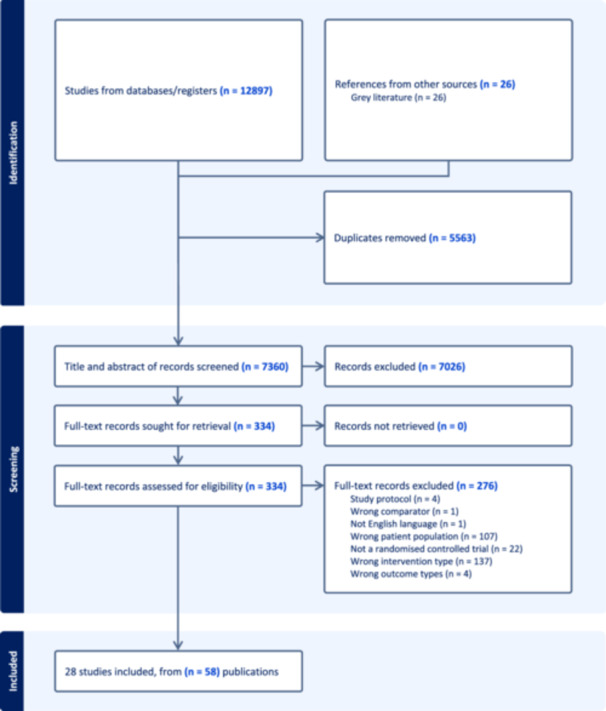
PRISMA flowchart (reproduced from Balasooriya Lekamge et al. (2025) as published in Springer Nature, under the CC BY license: https://creativecommons.org/licenses/).

All search results were imported into Endnote and duplicates removed. The results were then transferred into Covidence (Veritas Health Innovation, Melbourne, Australia), where further duplicates were removed. Each search result was then independently screened on Covidence by two reviewers (R.B.L., R.J., Y.Z., P.S., J. S.), initially by title and abstract and subsequently by full text article. Disagreements in the screening process were resolved by a third reviewer on the research team. The PRISMA flowchart outlining the screening process is presented in Figure 2. Two reviewers (R.B.L. and either R.J., Y.Z., P.S. or J.S.) completed risk‐of‐bias assessments independently for included studies using the Cochrane risk‐of‐bias tool for cluster randomised controlled trials (Eldridge et al. 2021). A third reviewer (DI) resolved disagreements in the risk‐of‐bias assessments. Data extraction for each included study was completed independently by two reviewers (R.B.L. and either R.J., Y.Z., P.S., J.S.), in alignment with a prespecified data extraction template. The theoretical underpinning of a whole‐school approach and previous, related systematic reviews (Langford et al. 2015; Goldberg et al. 2019) informed the construction of the template, which was piloted before use. The extracted data included information on study characteristics, initial training and ongoing support provided to those implementing interventions, and intervention components, the latter of which was subsequently mapped against the eight domains of a whole‐school approach. The narrative synthesis of this data and interactive EGM will serve as the focus of this article, with a separate article to present a synthesis of implementation assessment data and meta‐analyses. Given the different intervention foci, intervention strategies have been grouped by intervention type in the narrative synthesis (where possible) and in the interactive EGM to facilitate a more nuanced understanding of the current operationalisation of the eight domains within each intervention type.

## 5 Results

### 5.1 Results of Search

In total, 12,897 records were identified from searches of the scientific and grey literature. A total of 28 studies reported in 58 publications met the final inclusion criteria. The findings of the review are presented in three sections: (i) Section 1: Characteristics of the included studies; (ii) Section 2: Initial training and ongoing support provided to those involved in intervention implementation; and (iii) Section 3: Mapping intervention components against the eight domains of a whole‐school approach. An overview of the included studies is presented in Table 3. In the instance that several publications existed for the one study, the primary publication has been listed first in the table. A table of excluded studies is also presented in Supporting Information S2.

**Table 3:** Summary of included studies.

Study	Intervention type	Country	Target group	Duration (months)	Control	Theory	Initial training	Ongoing support	D1	D2	D3	D4	D5	D6	D7	D8
Allara et al. (2019)	Multiple risk behaviour	Italy	Grade 7 and 9	16	Inactive	—	X		X		X		X	X		
Andersen et al. (2015); Bast et al. (2019); Bast et al. (2015)	Smoking prevention	Denmark	Grade 7	12	Inactive	—			X				X	X		
Bond et al. (2004); Patton et al. (2006)	Multiple risk behaviour	Australia	Grade 8	36	Inactive	Health‐Promoting Schools Framework, Attachment Theory	X	X	X	X	X	X	X	X		
Bonell et al. (2018, 2020); Melendez‐Torres et al. (2022)	Multiple risk behaviour	England	Grade 7	36	Inactive	Health‐Promoting Schools Framework, Theory of Change, Theory of Human Functioning and School Organisation	X	X	X	X		X	X	X		
Bonnesen et al. (2023)	Multiple risk behaviour	Denmark	Grade 10	9	Inactive	Socio‐Ecological Model, Intervention Mapping Protocol	X		X				X	X	X	
Cross et al. (2016)	Cyberbullying prevention	Australia	Grade 8	24	Inactive	Socio‐Ecological Model, PRECEDE‐PROCEDE Model	X		X	X			X	X		
Cross et al. (2018)	Bullying prevention	Australia	Grade 8	24	Active	Socio‐Ecological Model, Attachment Theory, Social Cognitive Theory, Problem Behaviour Theory, Health‐Promoting Schools Framework	X	X	X	X		X	X	X		
De Vries et al. (2006) (Denmark); De Vries (2003) (Denmark)	Smoking prevention	Denmark	Grade 7	36	Inactive	Attitude‐Social Influence‐Self‐Efficacy Model			X	X	X		X	X		
De Vries et al. (2006) (Finland); De Vries (2003) (Finland); Vartiainen et al. (2007)	Smoking prevention	Finland	Grade 7	36	Inactive	Attitude‐Social Influence‐Self‐Efficacy Model	X	X	X	X	X		X	X		
Dray et al. (2017); Hodder et al. (2017, 2018)	Multiple risk behaviour	Australia	Grade 7–10	36	Inactive	Health‐Promoting Schools Framework	X	X	X	X		X		X	X	
Foshee et al. (1998)	Dating violence prevention	United States	Grade 8–9	5	Active	—	X		X	X	X			X	X	
Gorini et al. (2014); Carreras et al. (2016)	Smoking prevention	Italy	Grade 9	12	Inactive	Social Competence Approach, Social Influence Approach	X		X	X	X	X	X	X		
Hamilton et al. (2005)	Smoking prevention	Australia	Grade 9	24	Active	Health‐Promoting Schools Framework	X	X	X				X	X	X	X
Hunt (2007)	Bullying prevention	Australia	Grade 7–10	2.5	Active	—			X			X		X		
Johnson et al. (2017)	Mindfulness promotion	Australia	Grade 8	2.25	Inactive	—			X	X	X			X		
Kärnä et al. (2013)	Bullying prevention	Finland	Grade 7–9	12	Inactive	Social Cognitive Theory	X	X	X	X	X		X	X		
Larsen et al. (2019, 2023)	Multiple risk behaviour	Norway	Grade 11	36	Inactive	Self‐Determination Theory	X	X	X	X	X	X			X	
Malmberg et al. (2014); Malmberg et al. (2015)	Substance use prevention	The Netherlands	Grade 9	36	Inactive	Attitude‐Social Influence‐Self‐Efficacy Model	X		X				X	X	X	
Perry et al. (2003)	Multiple risk behaviour	United States	Grade 7	24	Inactive	—	X		X	X		X		X		
Perry et al. (2009); Bate et al. (2009)	Smoking prevention	India	Grade 8	24	Inactive	Social Influences Approach, Social Cognitive Theory	X	X	X	X	X			X		
Rahman et al. (1998)	Mental health literacy	Pakistan	Grade 8–12	4	Inactive	—			X	X	X	X		X		
Sawyer, Pfeiffer, et al. (2010); Sawyer, Harchak, et al. (2010); Spence et al. (2014)	Depression prevention	Australia	Grade 8	36	Active	Cognitive Behavioural Framework	X	X	X			X		X	X	
Schofield (2003)	Smoking prevention	Australia	Grade 7–8	24	Inactive	Health‐Promoting Schools Framework, Community Organisation Theory	X	X	X	X			X	X		
Shinde et al. (2020); Shinde et al. (2018); Singla et al. (2021)	Multiple risk behaviour	India	Grade 9	24	Active	Health‐Promoting Schools Framework	X	X	X	X	X	X	X		X	X
Skarstrand et al. (2014)	Alcohol prevention	Sweden	Grade 6 (aged 12)	24	Active	Biopsychosocial Model, Resiliency Model, Family Process Model	X		X	X				X		
Stevens et al. (2000)	Bullying prevention	Belgium	High school students	24	Inactive	Social Learning Theory	X	X	X			X	X	X		
Wen et al. (2010)	Smoking prevention	China	Grade 7–8	24	Active	Socio‐Ecological Model, PRECEDE‐PROCEED Model, Intervention Mapping	X		X	X	X	X	X	X		
Wolfe et al. (2009)	Dating violence prevention	Canada	Grade 9	3.75	Active	—	X		X			X		X		

Abbreviations: D1, school curriculum; D2, school social‐emotional environment; D3, school physical environment; D4, school governance and leadership; D5, school policies and resources; D6, school and community partnerships; D7, school health services; D8, government policy and resources.

### 5.2 Synthesis of Included Studies

#### 5.2.1 Characteristics of Included Studies

##### 5.2.1.1 Types of Interventions

**Figure 3:**
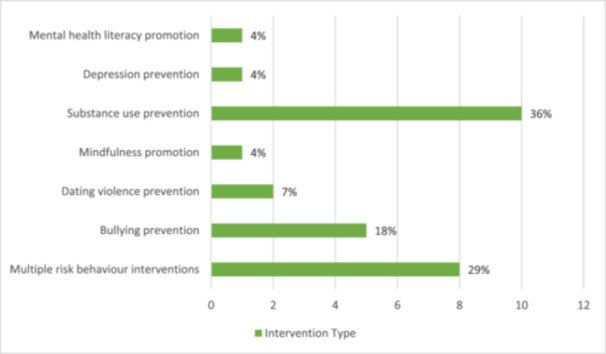
Distribution of studies per intervention type.

Most of the studies (10 of 28 studies) involved interventions that classified as substance use prevention (Table 3), with eight of these focused on smoking prevention, one on alcohol prevention, and one on the combined prevention of alcohol, tobacco and marijuana use. Multiple risk behaviour interventions were the second most common intervention type (8 of 28 studies), targeting multiple outcome domains including positive mental health, mental disorders, bullying, aggression and substance use through the single intervention. Four of these had the concurrent aim of modifying the school climate and physical health behaviours, including physical activity, sleep and diet. The remaining 10 studies were classified as follows: bullying prevention (5 studies), dating violence prevention (2 studies), depression prevention (1 study), mental health literacy promotion (1 study) and mindfulness promotion (1 study). The percentage of studies for each intervention type is summarised in Figure 3.

##### 5.2.1.2 Study Design

All studies employed cluster randomised controlled designs. Studies almost exclusively randomised clusters by school‐level, except for Johnson et al. (2017) who randomised clusters by class‐level.

##### 5.2.1.3 Target Group

**Figure 4:**
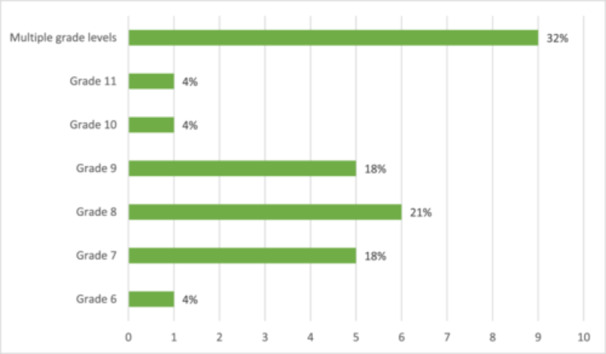
The distribution of studies addressing each grade level.

The majority of studies (18 of 28 studies) involved samples in a single grade level, most frequently grade seven (5 studies) or grade eight (6 studies). Eight studies explicitly involved samples across multiple grade levels (Table 3). Figure 4 portrays a visual representation of the distribution of studies targeting each grade level.

##### 5.2.1.4 Country of Study

Nine studies were set in Australia. Three studies were set in Denmark, and two studies were set in each of Italy, the United States, India and Finland. There was one study set in each of England, The Netherlands, China, Norway, Canada, Belgium, Pakistan and Sweden (Table 3).

##### 5.2.1.5 Duration of Intervention

Interventions ranged from 9 weeks to 3 years in duration (Table 3). There were eight studies that spanned 3‐years, 10 studies that spanned 2‐years, 1 study that spanned 16‐months, and 9 studies that spanned 1‐year or less in duration.

##### 5.2.1.6 Control Condition

Nineteen studies described ‘school‐as‐usual’ as the control condition, while nine explicitly referenced that the control group had implemented one or more initiatives that overlapped with the intervention's aims (denoted as ‘active’ in Table 3). These included the same or similar curriculum components to the intervention group, the same community‐based initiative as the intervention group, bullying prevention policies and related disciplinary procedures. Governmental mandates were the most common reason cited for these activities in the control group.

##### 5.2.1.7 Theoretical Basis

A wide variety of theories were referenced by studies (Table 3). Some described that their intervention was based on a single theory, others on multiple theories, and eight studies did not reference any theory. The most commonly referenced theories were the Health‐Promoting Schools Framework (7 studies), the socio‐ecological model (4 studies), social cognitive theory (3 studies), the attitude‐social influence‐self‐efficacy model (3 studies), attachment theory (2 studies), intervention mapping (2 studies), the social influences approach (2 studies) and the PRECEDE‐PROCEED model (2 studies). The following theories were each referenced by one study: theory of change, theory of human functioning and school organisation, problem behaviour theory, the cognitive behavioural framework, social competence approaches, community organisation theory, self‐determination theory, social learning theory, the biopsychosocial model, the resiliency model and the family process model.

##### 5.2.1.8 Youth Participation in Whole‐School Interventions

Youth were explicitly involved in intervention design (in some capacity) in 8 studies (29%). Several referenced the co‐development of whole‐school interventions with youth (Bonell et al. 2018; Cross et al. 2016, 2018; Kärnä et al. 2013; Shinde et al. 2020). Bonnesen et al. (2023) conducted a comprehensive needs assessment with youth to inform the design of their whole‐school intervention, while three other studies consulted youth through qualitative research to explore the appropriateness of the proposed whole‐school intervention strategies before their implementation in the randomised controlled trial (Bonnesen et al. 2023; Perry et al. 2009; Wen et al. 2010). All studies involved students in the evaluation stage, 61% in the implementation of intervention strategies and 39% in the planning of intervention strategies. These are discussed in more detail in Section 5.2.4.

#### 5.2.2 Study Quality

**Figure 5:**
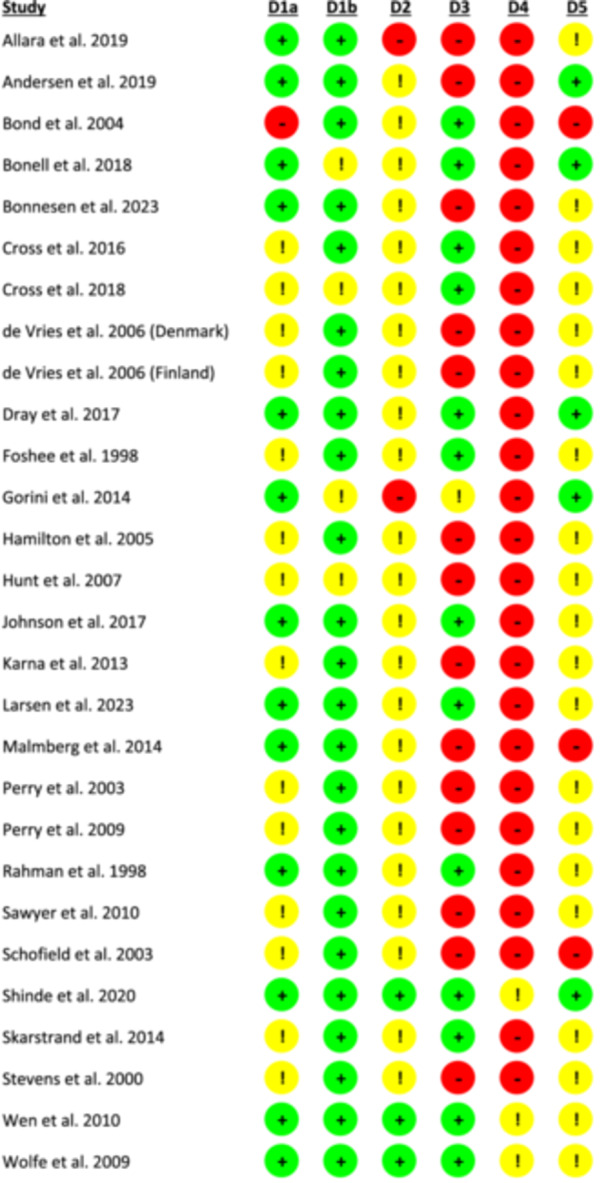
Risk‐of‐bias assessments for each included study (reproduced from Balasooriya Lekamge et al. (2025) as published in Springer Nature, under the CC BY license: https://creativecommons.org/licenses/). D1a and 1b assess selection bias, D2 performance bias, D3 attrition bias, D4 detection bias and D5 reporting bias. Green indicates low risk, yellow indicates unclear risk and red indicates high risk.

Most studies (14 of 28 studies) were classified as unclear risk of bias regarding random sequence generation (Domain 1a), frequently because they identified themselves as randomised controlled trials without sufficient explanation of the method used to generate a truly random sequence. Most studies (24 studies) were classified as low risk regarding allocation concealment (Domain 1b), by clearly detailing that randomisation of clusters occurred at the commencement of the trial. For performance bias (Domain 2), the majority of studies (23 studies) were classified as unclear risk, commonly because these studies did not explicitly reference whether deviations from the study protocol occurred. Most studies (14 studies) were classified as low risk for attrition bias (Domain 3), including all clusters and nearly all participants within each cluster in statistical analyses. The vast majority of studies (25 studies) were classified as high risk for detection bias (Domain 4), due to study participants serving as outcome assessors alongside the use of self‐reporting measures. For reporting bias (Domain 5), the majority of studies (20 studies) were classified as unclear risk, because these studies did not reference a study protocol. Figure 5 provides an overview of the risk‐of‐bias assessments for each study, with detailed justification for each judgement provided in Supporting Information S3.

#### 5.2.3 Initial Training and Ongoing Support Provided to Those Involved in Intervention Implementation

##### 5.2.3.1 Initial Training

###### 5.2.3.1.1 What Was Implemented?

A total of 23 studies (82%) included initial training (Table 3). The content covered in training sessions varied by study and canvassed the following areas: (i) an introduction to the goals and rationale for the intervention, intervention components and evaluation design; (ii) training teachers to deliver the curriculum component, including up‐skilling teachers in interactive teaching strategies; (iii) facilitating acquisition of the relevant knowledge and skills required for intervention delivery; (iv) training school action teams, welfare staff and peer leaders to deliver whole‐school components; and (v) training staff to troubleshoot potential challenges in implementation.

###### 5.2.3.1.2 How Was It Implemented?

Four studies included 2–4‐hour training sessions (Bonell et al. 2018; Cross et al. 2018; Gorini et al. 2014; Perry et al. 2003), 10 studies described 6–12‐hour sessions that were presented either as a day‐long training workshop or in smaller chunks (Allara et al. 2019; Bond et al. 2004; Bonnesen et al. 2023; Cross et al. 2016, 2018; Hamilton et al. 2005; Perry et al. 2009; Sawyer, Pfeiffer, et al. 2010; Wen et al. 2010; Wolfe et al. 2009), while 8 studies described more intensive requirements: a 2‐day training workshop (Kärnä et al. 2013; Larsen et al. 2019; Sawyer, Pfeiffer, et al. 2010), a 3‐day training workshop for selected staff (Bonell et al. 2018), 20 hours' worth of training (De Vries et al. 2006 (Finland); Foshee et al. 1998), 25 hours' worth of training (Stevens et al. 2000) and a week‐long training workshop (Shinde et al. 2020).

###### 5.2.3.1.3 Who Was Involved?

Initial training was largely provided by research staff; however, in two studies, public health professionals and lead teachers provided training (Allara et al. 2019; Wolfe et al. 2009). Teachers most commonly received training (Allara et al. 2019; Bond et al. 2004; Cross et al. 2016, 2018; Dray et al. 2017; Gorini et al. 2014; Hamilton et al. 2005; Malmberg et al. 2014; Perry et al. 2009; Sawyer, Pfeiffer, et al. 2010; Shinde et al. 2020; Skarstrand et al. 2014; Wen et al. 2010; Wolfe et al. 2009), followed by school action teams in four studies (Bonnesen et al. 2023; Cross et al. 2018; Larsen et al. 2023; Sawyer, Pfeiffer, et al. 2010), peer leaders in four studies (Cross et al. 2016; Larsen et al. 2023; Perry et al. 2009; Skarstrand et al. 2014), welfare staff including school nurses and pastoral care staff in four studies (Cross et al. 2016; Sawyer, Pfeiffer, et al. 2010; Shinde et al. 2020; Wen et al. 2010), all school staff in two studies (Bonell et al. 2018; Cross et al. 2018), and police officers who delivered the curriculum (Perry et al. 2003) and year level coordinators (Sawyer, Pfeiffer, et al. 2010), in one study each.

##### 5.2.3.2 Ongoing Support

###### 5.2.3.2.1 What Was Implemented?

Overall, 13 studies (46%) provided ongoing support (Table 3). The types of support provided varied by study, comprising (i) boosting motivation of school staff to implement the programme; (ii) providing ongoing professional development for staff; (iii) assisting schools in identifying student and staff needs and selecting appropriate strategies to implement; (iv) monitoring implementation, providing supervision and individualised performance feedback; and (v) assisting schools to overcome possible obstacles and receiving school‐initiated escalations for support.

###### 5.2.3.2.2 How Was It Implemented?

In certain schools, ongoing support was provided through school‐based meetings that occurred weekly (Bond et al. 2004), monthly (Shinde et al. 2020; Sawyer, Pfeiffer, et al. 2010), three times per year (Kärnä et al. 2013), four times per year (Cross et al. 2018), or through the presence of an intervention officer who was available at schools 1 day per week (Dray et al. 2017). In other studies, ongoing support was delivered through phone and/or email contact (Hamilton et al. 2005; Larsen et al. 2023), or in the case of Sawyer, Pfeiffer, et al. (2010), weekly phone contact supplemented the monthly school‐based meetings.

###### 5.2.3.2.3 Who Was Involved?

A school liaison officer or team were typically derived from research project staff, with experience working in, or qualifications related to, the education or health sector. In many studies, it was unclear who these staff supported within the school setting; however, two studies described that support was provided directly to school action teams (Bond et al. 2004; Sawyer, Pfeiffer, et al. 2010).

#### 5.2.4 Mapping Intervention Components Against the Eight Domains of a Whole‐School Approach

**Figure 6:**
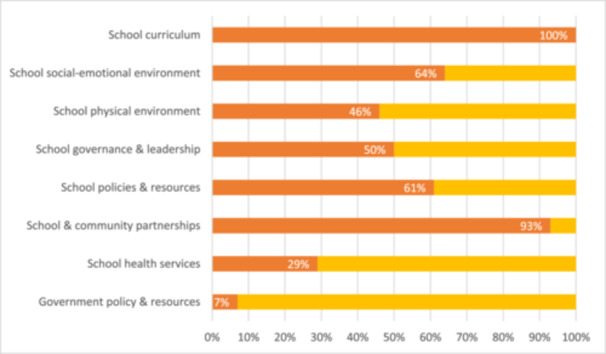
The percentage of studies addressing each of the eight domains of a whole‐school approach.

The frequency with which the interventions implemented by studies addressed the eight domains of a whole‐school approach is summarised in Figure 6.

##### 5.2.4.1 Domain 1: School Curriculum

###### 5.2.4.1.1 What Was Implemented?

**Figure 7:**
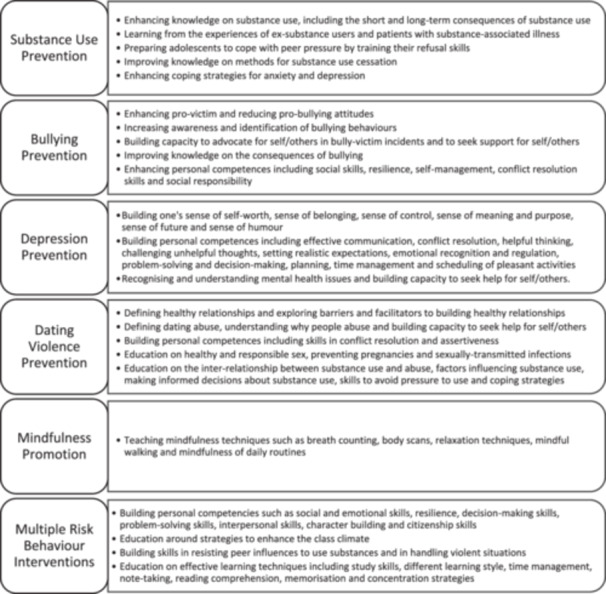
Explicit educational content summarised by intervention type.

Figure 7 presents the explicit educational content as summarised by intervention type. Four studies outlined the physical resources provided to those delivering the intervention curriculum (Bonell et al. 2018; Sawyer, Pfeiffer, et al. 2010; Johnson et al. 2017; Wolfe et al. 2009). These included detailed lesson plans, intervention manuals, presentation slides, individual student workbooks and resources for all activities (such as role play exercises, worksheets, DVD and video materials).

###### 5.2.4.1.2 How Was It Implemented?

The duration of curriculum‐based sessions varied significantly across studies, ranging from a one‐off 2‐hour session (Gorini et al. 2014; Hunt 2007) to a 21‐session curriculum comprising of 75‐min lessons (Wolfe et al. 2009). In the majority of multi‐year studies, the curriculum component was implemented in each year of the study. In two studies, the curriculum component was delivered entirely through online modules (Malmberg et al. 2014; Cross et al. 2016), with one study citing that the rationale for this was that teachers reported limited self‐efficacy to teach about cyber‐safety. Many of the studies referenced implicit education and detailed the use of inclusive, participatory methods to engage students, including quizzes, case studies, small group activities, role plays, panel discussions, self‐reflection activities, virtual learning environments, as well as chat rooms and forums to discuss relevant topics and exchange opinions.

###### 5.2.4.1.3 Who Was Involved?

School teachers delivered the intervention curriculum in the vast majority of studies. Peer leaders collaborated with teachers in delivering the intervention curriculum in three studies (Perry et al. 2009; Skarstrand et al. 2014; Larsen et al. 2023). For example, in Perry et al. (2009), a teacher‐led discussion opened and closed each session, with trained peers facilitating small group activities for the main part of the session. School nurses were involved in delivering the curriculum in two studies (De Vries et al. 2006 (Finland); Wen et al. 2010), and counsellors (Shinde et al. 2020), researchers (Johnson et al. 2017) and police officers (Perry et al. 2003) in one study each.

##### 5.2.4.2 Domain 2: School Social‐Emotional Environment

###### 5.2.4.2.1 Peer Leadership and Support Programmes

Peer leadership and support programmes were the most common strategy in this domain (11 studies). As mentioned above, peer leaders collaborated with teachers in delivering the intervention curriculum in three studies. Peer‐led activities featured in four studies: in Cross et al. (2016), peers were trained to lead at least three whole‐school activities to encourage students' positive use of technology; in Gorini et al. (2014), self‐selected peers conducted two meetings with students on the positive and negative aspects of smoking and led a creative writing session on smoking; in Perry et al. (2009), programmes featured peer‐led activism outside of the classroom; and in Perry et al. (2003), elected and trained peers led classroom activities designed as part of a teenage magazine, which related to the influences from social groups, media and role models and the skills required to manage these. Peer support programmes featured in five studies: peers supported students in dealing with smoking issues (Schofield 2003); teacher‐selected prosocial and high‐status peers supported students who were victim to bullying through inviting them to activities and trying to make others stop bullying (Kärnä et al. 2013); peers welcomed new students on the first day of school, conveyed information about class and school gatherings and created meeting points for socialisation (Larsen et al. 2023); and general peer support programmes (Bond et al. 2004, Dray et al. 2017). In Shinde et al. (2020), monthly meetings were held between students elected from each class and staff leading the intervention, to discuss student concerns and the health topic of the month, develop an action plan and assist in coordinating the activities of the whole‐school intervention (such as assembly‐level activities and competitions).

###### 5.2.4.2.2 Staff‐Led Strategies

Staff‐led strategies to improve school ethos included teachers implementing mindfulness practice outside of formal lessons (Johnson et al. 2017); staff delivering restorative practices such as circle time to build relationships among students and restorative conferences to address more serious incidents (Bonell et al. 2018); non‐curriculum programmes targeting protective factors outside the classroom such as through school assemblies, camps and welfare days (Dray et al. 2017); a schoolwide ceremony to celebrate the ‘World No Tobacco Day’, encouragement for students to sign a public commitment not to smoke and distribution of a booklet reiterating themes covered in the intervention curriculum (Wen et al. 2010).

###### 5.2.4.2.3 Student Magazines

Student magazines were employed by two studies to reinforce the intervention's key themes. In Cross et al. (2018), a magazine was sent to students with information on the organisational and academic changes they could expect in the transition from primary to secondary school and tips to help them maintain old and build new friendships. In De Vries et al. (2006) (Finland), tabloid‐style newsletters with explanations from peer models on their decision to be non‐smokers and how to refrain from smoking were circulated to adolescents.

###### 5.2.4.2.4 Competitions

Competitions were another strategy employed by four studies, with the aim of reinforcing key themes of the intervention. Foshee et al. (1998) hosted a poster contest that addressed the themes introduced by the curriculum component, with posters hung in classrooms and voted on by students, with the top three posters earning a cash prize; Wen et al. (2010) hosted anti‐smoking essay, presentation and poster contests; Rahman et al. (1998) hosted annual speech, essay‐writing and poster‐painting contests with mental health themes; and De Vries et al. (2006) (Finland) hosted a non‐smoking competition.

###### 5.2.4.2.5 Other Strategies

Other less commonly employed strategies included postcards being sent to students. In De Vries et al. (2006) (Denmark), postcards used positive non‐smoking frames, and in Perry et al. (2003), postcards explained the ways in which the tobacco industry targeted youth. Two studies involved students participating in a theatre production to highlight key messages in the intervention (Foshee et al. 1998; Perry et al. 2003). For example, in Foshee et al. (1998), the play was about how an adolescent victim of dating violence seeks help with her violent relationship and highlights various mediating variables related to help‐seeking.

##### 5.2.4.3 Domain 3: School Physical Environment

###### 5.2.4.3.1 Posters

The most frequently employed strategy in this domain were posters displayed in the classroom or broader school environment (10 studies). Posters were commonly used to reinforce ideas introduced by the interventions. For example, in Perry et al. (2009), posters corresponded with classroom activity themes; De Vries et al. (2006) (Denmark) used anti‐smoking posters, Johnson et al. (2017) implemented posters reinforcing their four‐step mindfulness practice; Rahman et al. (1998) used posters displaying mental health slogans; Larsen et al. (2023) included posters involving guidelines for a good psychosocial class environment; Kärnä et al. (2013) implemented posters reminding students and staff about the anti‐bullying intervention; and in Bond et al. (2004), classroom rules were negotiated between teachers and students and displayed as a poster in each classroom. In three studies, student‐created posters (frequently the winning posters) were derived from poster contests and displayed in schools; two related to anti‐smoking content (De Vries et al. 2006 (Finland); Wen et al. 2010) and one to dating violence prevention (Foshee et al. 1998).

###### 5.2.4.3.2 Interactive Information Boards

Interactive information boards were described in two studies. In Shinde et al. (2020), a monthly wall magazine was implemented, with content submitted by students and school staff posted on the wall following approval by the teachers and counsellors delivering the intervention. In Wen et al. (2010), students created an information area on the association between smoking and health on the blackboard of each classroom.

###### 5.2.4.3.3 Other Strategies

Less common strategies included (i) improving non‐smoking signage (Gorini et al. 2014; Wen et al. 2010); (ii) providing bright vests for recess supervisors to enhance their visibility and indicate bullying was taken seriously at the school (Kärnä et al. 2013); (iii) an anonymous letterbox for students to raise their concerns and questions (Shinde et al. 2020); and (iv) promoting healthy foods in vending machines and canteens (Allara et al. 2019).

##### 5.2.4.4 Domain 4: School Governance and Leadership

###### 5.2.4.4.1 School Action Teams

Two foci existed for this domain: the organisation of school action teams, and professional development opportunities. With relation to the former, 11 studies setup school action teams (Bond et al. 2004; Bonell et al. 2018; Cross et al. 2018; Dray et al. 2017; Gorini et al. 2014; Larsen et al. 2023; Perry et al. 2003; Sawyer et al. 2010; Shinde et al. 2020; Wen et al. 2010; Wolfe et al. 2009). In one study, the school action team consisted of staff alone, but more commonly, it also included students (6 studies), parents (1 study) and/or external personnel (5 studies). External personnel comprised of research team members in three studies and external facilitator staff from community organisations in two studies. The staff involved in school action teams included year level coordinators, welfare staff, teachers and senior executive staff, with the school principal involved in four studies.

Across the studies, the school action team's role included (i) reviewing the needs assessment data from their student cohort; (ii) coordinating prescribed intervention strategies or selecting evidence‐based strategies according to the school's needs and capacity; (iii) revising or newly implementing school policies; (iv) monitoring the progress of intervention strategies; and (iv) ensuring local adaptation of strategies to suit the school's student cohort. Needs assessment data were derived from various means; for example, issues submitted by students in an anonymous letterbox (Shinde et al. 2020); an audit conducted by research staff on the school's current structures, policies, programmes and practices related to school wellbeing (Sawyer, Pfeiffer, et al. 2010); and anonymous findings from the school's baseline survey (collected by research staff) to understand the school's local needs (Bond et al. 2004; Bonell et al. 2018).

The frequency of school action team meetings was referenced by four studies, occurring either monthly (Sawyer, Pfeiffer, et al. 2010), twice per term (Dray et al. 2017; Bonell et al. 2018), or twice per year (Shinde et al. 2020). Four studies specifically referenced that the school action team had received training for their role.

###### 5.2.4.4.2 Staff Professional Development

In relation to the staff professional development strategy, this commonly involved increasing awareness on the levels of bullying (as reported by students in the baseline survey) and the nature of bullying in schools, as well as establishing consistent approaches to bullying prevention and effective disciplinary practices among staff for managing bullying incidents (Cross et al. 2018; Shinde et al. 2020; Hunt 2007; Stevens et al. 2000). In Rahman et al. (1998), a short training course was provided for teachers on common mental illnesses found in the community. Only Hunt (2007) provided further details on who provided professional development sessions, and specified that the training was delivered to school staff by the research team in regular staff meetings.

##### 5.2.4.5 Domain 5: School Policies and Resources

###### 5.2.4.5.1 Anti‐Substance Use Policies

Most of the studies described policies and procedures in relation to substance use (Allara et al. 2019; Andersen et al. 2015; De Vries et al. 2006 (Denmark); De Vries et al. 2006 (Finland); Gorini et al. 2014; Hamilton et al. 2004; Malmberg et al. 2014; Schofield 2003; Shinde et al. 2020; Wen et al. 2010). Eight studies described smoking bans for students (Allara et al. 2019; Andersen et al. 2015; De Vries et al. 2006 (Denmark); De Vries et al. 2006 (Finland); Gorini et al. 2014; Hamilton et al. 2004; Schofield 2003; Wen et al. 2010), one study described prohibition of alcohol use during school events (Allara et al. 2019), and two studies described broader anti‐substance use policies (Malmberg et al. 2014; Shinde et al. 2020). In Allara et al. (2019) and Andersen et al. (2015), smoking bans encompassed teachers as well; however, in Andersen et al. (2015), the original study design had to be adapted to include an outdoor area for teachers to smoke. Wen et al. (2010) required an explicit commitment on behalf of the school to support an anti‐smoking initiative, while De Vries et al. (2006) (Finland) provided smoking cessation resources for school personnel. Gorini et al. (2014) provided a more detailed description of their anti‐smoking policy, requiring that schools included clear indication of non‐smoking areas, sanctions and enforcement surveillance, with the school working group monitoring enforcement of these procedures.

###### 5.2.4.5.2 Anti‐Bullying Policies

The second most common strategy in this domain included schools implementing or revising their anti‐bullying policies and procedures (Bond et al. 2004; Cross et al. 2016, 2018; Kärnä et al. 2013; Shinde et al. 2020; Stevens et al. 2000). Two studies provided a more detailed description of what this involved. In Stevens et al. (2000), the anti‐bullying policy had to include a clear definition of bully/victim problems and clearly convey to students that bullying would not be tolerated. Contracting was used between the teacher and bully, whereby teachers enforced the immediate consequences of bullying behaviour, encouraged empathy in the bully towards the victim, and encouraged the bully to make up for their behaviour by doing something for the victim or class group. Intensive emotional support was also provided to the victim, alongside discussion of strategies to handle bullying incidents. In Kärnä et al. (2013), a small team of school staff, along with the classroom teacher, reviewed each case of bullying raised to examine whether the case constituted bullying. In the event that it did, staff held individual discussions with the victim to hear about their experience and reaffirm the school's intention to end the bullying. Individual discussions were then held with the bully, without prior notice, with one of two strategies adopted: (i) to convey that the bullying must stop immediately, or (ii) to share their concerns about the victim and invite the bully to provide suggestions on how the situation could be improved. Follow‐up meetings were then held with the bully and victim to ensure that the bullying had stopped permanently.

###### 5.2.4.5.3 Other Policy Examples

Policies less frequently implemented included those to support the use of restorative practices (Bonell et al. 2018) and those related to stress (Bonnesen et al. 2023). In the latter, schools had to establish guidelines, practices and a clear action plan for stress management in students, including annual coursework plans that detailed assignment due dates and the expected amount of time to complete assignments for each class.

###### 5.2.4.5.4 Considerations for Policy Implementation

Eight studies detailed who was involved in implementing and revising policies. This included school action teams in two studies (Malmberg et al. 2014; Gorini et al. 2014), the pastoral care team in one study (Cross et al. 2016), school staff in one study (Stevens et al. 2000), and a combination of parents, students, teachers and the school executive team, often followed by approval from the school action team or school authorities, in the remaining four studies (Allara et al. 2019; Shinde et al. 2020; Bonell et al. 2018; Cross et al. 2018). Other studies detailed the communication of policies to the relevant cohort; for example, in Shinde et al. (2020), this was achieved through announcements at school assembly and on school noticeboards.

##### 5.2.4.6 Domain 6: School and Community Partnerships

Three major activities emerged: (i) activities within the schooling community; (ii) activities between the school and local community; and (iii) a combination of both activities.

###### 5.2.4.6.1 Activities Within the Schooling Community

**Figure 8:**
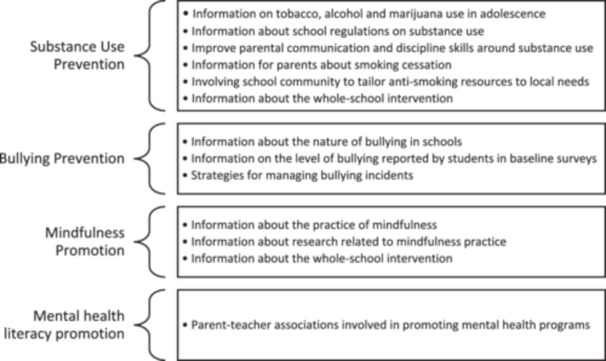
Parental meeting content summarised by intervention type.

With relation to (i), meetings with parents were held in nine studies (De Vries et al. 2006 (Denmark); De Vries et al. 2006 (Finland); Hunt 2007; Johnson et al. 2017; Malmberg et al. 2014; Rahman et al. 1998; Schofield 2003; Skarstrand et al. 2014; Stevens et al. 2000). Parental meetings were described to be 60–90‐min in duration; commonly involved one‐off sessions but could include up to 12 sessions (Skarstrand et al. 2014); were conducted by the research team after‐hours at school; and were supplemented by physical resources for parents in six of the nine studies (De Vries et al. 2006 (Denmark); De Vries et al. 2006 (Finland); Hunt 2007; Johnson et al. 2017; Malmberg et al. 2014; Schofield 2003). The content covered in parental meetings is summarised by intervention type in Figure 8.

**Figure 9:**
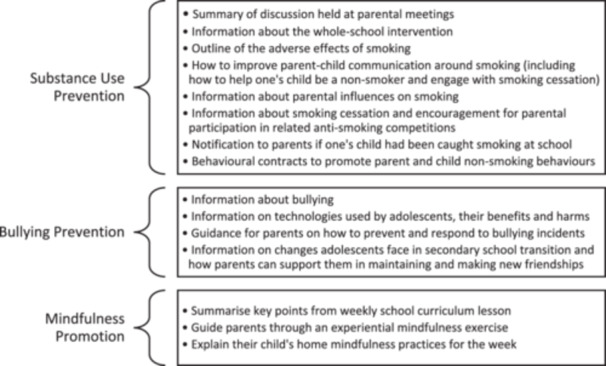
Informational resources content summarised by intervention type.

Informational resources were another common strategy to engage parents and were described by 16 studies (Andersen et al. 2015; Cross et al. 2016, 2018; De Vries et al. 2006 (Denmark); De Vries et al. 2006 (Finland); Dray et al. 2017; Foshee et al. 1998; Hamilton et al. 2004; Hunt 2007; Johnson et al. 2017; Kärnä et al. 2013; Malmberg et al. 2014; Perry et al. 2003; Perry et al. 2009; Schofield 2003; Wen et al. 2010). These commonly included booklets and pamphlets (7 studies), newsletter items (6 studies), formal letters (3 studies), smoke‐free contracts (2 studies), online resources including specially designed YouTube clips (2 studies), postcards containing short behavioural messages (2 studies), and take‐home activities for students to complete with parents (1 study). While the content included in the informational resources substantially overlapped with the content delivered through the parental meetings, subtle differences existed and therefore this content is summarised separately in Figure 9.

To expand upon an example of a behavioural contract implemented, in Wen et al. (2010), a detailed contract required of parents that they not smoke at home, try quit smoking, decrease access to cigarettes at home, limit their child's spending money, communicate with children about not smoking, supervise their smoking behaviours, and assist them to refuse cigarettes; and required of adolescents that they say ‘no’ to offered cigarettes and help smoking parents quit, with the signed contract posted at home.

###### 5.2.4.6.2 Activities Between the School and Local Community

Twelve studies addressed activities between the school and local community, comprising of a diverse array of intervention strategies. Three studies involved research teams providing schools with data to outline their local psychosocial needs (Bond et al. 2004; Bonell et al. 2018; Sawyer et al. 2010). Two of these studies achieved this through provision of the school's baseline survey data compared with other intervention or control schools, and the remaining study undertook an audit of the school's current policies and practices (Sawyer, Pfeiffer, et al. 2010). Six studies encouraged greater collaboration with community organisations: Perry et al. (2003) setup neighbourhood action teams to address neighbourhood issues related to drug use and violent behaviours; Dray et al. (2017) encouraged promotion and engagement of local community clubs, groups and organisations in schools; De Vries et al. (2006) (Denmark) provided schools with an inventory of youth clubs and information for community youth leaders on how to talk to young people about smoking; and Wolfe et al. (2009) sent schools a manual describing ways to involve students in school and community violence prevention activities. In Gorini et al. (2014), intervention students undertook a 4‐hour excursion to a health promotion centre, engaging in various interactive activities such as a lab‐based session showcasing the physiology of the respiratory system and the harmful ingredients found in cigarettes. In Bonnesen et al. (2023), university students from health‐related degrees facilitated two 90‐min workshops during school hours, to inspire high school students to create and implement new activities to build a sense of community and promote physical activity at their school. Teachers were then encouraged to support students in honing their ideas, apply for funding (where relevant) and implement the proposed activities.

Other intervention strategies involved Schofield (2003) sending letters to tobacco retailers, while Wen et al. (2010) secured commitments from grocery stores near certain intervention schools to not sell cigarettes to school students. The media was also engaged, with De Vries et al. (2006) (Denmark) employing 2 months of non‐smoking commercials on television and Wen et al. (2010) featured in an anti‐smoking television news report showcasing the exhibition of student‐made posters, animal experiments and interviews with students and research staff. In Bonnesen et al. (2023), an app was co‐developed by the research team and an app company, including articles about stress, its signs and symptoms; quizzes debunking myths about stress; techniques to prevent and reduce stress; time management techniques and access to an 8‐week text messaging service that provided additional tips to manage stress. In De Vries et al. (2006) (Finland), school dentists informed students on the hazards of smoking and how smoking affects their gums and teeth during routine dental appointments. This study also trained camp leaders at parish confirmation camps frequently attended by adolescents to motivate students to undertake non‐smoking activities.

###### 5.2.4.6.3 Activities Targeting the Schooling Community and Local Community

Two studies involved activities targeting both the school and local communities. This included walking activities between families, the local and school community in Allara et al. (2019). In Sawyer, Pfeiffer, et al. (2010), community forums were held that provided information about the nature and prevalence of mental health issues among students, risk and protective factors, and help‐seeking strategies for students, family members and friends. These were held during the school day or in the evening, and involved school action teams, project facilitators, staff, students and local community members.

##### 5.2.4.7 Domain 7: School Health Services

Three broad intervention strategies emerged in this domain: the provision of counselling services, establishing or improving referral pathways to health services, and upskilling service providers to work with adolescents.

###### 5.2.4.7.1 Counselling Services

The first strategy was to increase the awareness and accessibility of counselling services (6 studies). Dray et al. (2017) promoted engagement with health services in the school; Shinde et al. (2020) offered counselling services that students could voluntarily engage with; Bonnesen et al. (2023) mandated half‐year counselling sessions for all students; Foshee et al. (1998) offered weekly support groups for adolescent victims of dating abuse; and Hamilton et al. (2005) involved school‐based nurses offering counselling services to support students in reducing cigarette smoking. The most comprehensive of these services was described in Larsen et al. (2023), where a Mental Health Support Team comprising of counsellors, school nurses and follow‐up services was established; services and staff were re‐organised to be situated in the same location to improve their accessibility; the health and wellbeing of all students was mapped through the Kidscreen assessment, with follow‐up of at‐risk students and those demonstrating signs of absenteeism; and support was provided to teachers working with at‐risk students.

###### 5.2.4.7.2 Referral Pathways

The second of these strategies was to improve the referral pathways of students to health services. Both Shinde et al. (2020) and Dray et al. (2017) described establishing referral pathways for students; Malmberg et al. (2014) provided a training session for school staff on how to recognise problematic substance use in adolescents and how to support these adolescents both inside and outside of the school setting; Sawyer, Pfeiffer, et al. (2010) required school staff to attend a half‐day training that outlined mental health issues, support services and methods of referral, required schools to develop a mental health charter and assess their referral practices to community organisations.

###### 5.2.4.7.3 Upskilling Service Providers

The final of these strategies was to upskill service providers to work with adolescents. Foshee et al. (1998) offered 20 3‐hour workshops to community‐based health providers who worked with adolescents on the cognitive factors influencing help‐giving, and provided training to crisis‐line volunteers on how to respond to calls from adolescents. Hamilton et al. (2005) provided school‐based nurses with 3 hours of training to provide counselling services to help students reduce smoking.

##### 5.2.4.8 Domain 8: Government Policies and Resources

Two studies explicitly referenced government support, by means of a government‐mandated curriculum that was implemented in both intervention and control schools. In Shinde et al. (2020), this took the form of 16 hours' worth of classroom‐based sessions delivered by teachers each academic year, on growing up, establishing positive and responsible relationships, gender and sexuality, prevention of sexually transmitted infections, and substance use. In Hamilton et al. (2005), this involved 7 hours' worth of a smoking education curriculum, delivered by teachers who were trained state‐wide.

## 6 Discussion

### 6.1 Summary of Main Results

Our EGM makes an important contribution to the literature by providing a comprehensive synthesis of how whole‐school interventions promoting mental health and preventing risk behaviours in adolescence operationalise the eight domains of the Health‐Promoting Schools Framework. This enables appraisal of current practice against the recommendations of the Framework and identification of critical evidence gaps to facilitate the optimal translation of theory into practice. Our EGM identifies significant variability in how frequently whole‐school interventions address each of the eight domains (Figure 6). Variability ranges from 7% of studies including interventions that explicitly referenced the government policies and resources domain, to 100% of studies including interventions that addressed the school curriculum domain. While previous systematic reviews had found the school and community partnerships domain to be the weakest (Goldberg et al. 2019; Langford et al. 2015), our EGM identified that the government policies and resources domain was the least frequently referenced (Figure 6). A possible explanation for this discrepancy is that the government policies and resources domain had not been clearly demarcated in previous iterations of the Health‐Promoting Schools Framework; hence was likely not accounted for by previous reviews. In the setting of our EGM, it is difficult to ascertain whether this weak representation reflects a true lack of governmental support for whole‐school interventions in study contexts, or whether support existed, but had not been explicitly detailed by studies. In the event of the former, this raises concern around the equity of access to and sustainability of whole‐school interventions, given that a lack of funding and staff capacity are established barriers to the implementation of school‐based interventions (Cefai et al. 2021). Overall, an average of 4.5 of the 8 domains were addressed by the whole‐school interventions implemented by the included studies, with interventions occasionally demonstrating only passive efforts, such as posters, to address particular domains. This begs the question: *how whole‐school are whole‐school interventions*?

Despite the different intervention types included in our EGM, there was a clear overlap in the strategies implemented by interventions. This nexus can readily be observed through the school curriculum domain, where health literacy around substance use was included in not only substance use prevention, but also in dating violence prevention and multiple risk behaviour interventions. Health literacy relating to anxiety and depression was featured not only in depression prevention, but also in substance use prevention. Finally, developing personal competences served as an undercurrent for the majority of intervention types. This phenomenon has been highlighted in the literature (Boustani et al. 2015; Chorpita and Daleiden 2009; Skeen et al. 2019), which advocates moving away from single‐issue prevention programmes to identifying a core set of strategies to prevent a spectrum of youth mental health and behavioural issues (Barry et al. 2017; Boustani et al. 2015; Goldberg et al. 2019). This approach is underpinned by an aggregation of risk and protective factors that are common to a wide range of youth mental health and behavioural issues (Boustani et al. 2015). Such approaches would additionally relieve the pressure on schools in having to select from what appear to be competing priorities (Boustani et al. 2015). For example, in their review of universal school‐based interventions promoting mental health and preventing risk behaviours, Skeen et al. (2019) identified seven core intervention strategies that consistently predicted intervention success across a broad range of youth mental health and behavioural outcomes. These intervention strategies included developing interpersonal skills, emotional regulation, mindfulness, problem‐solving skills, drug and alcohol education, stress management and assertiveness training. However, because their review included universal interventions more broadly, these findings may not be generalisable to whole‐school interventions. The adoption of a core set of intervention strategies is consistent with the theoretical basis of a whole‐school approach, which champions a departure from a reductionist focus on single issues, risk factors and linear causality, towards a more holistic approach to health promotion and prevention (Samdal and Rowling 2012).

Few studies referenced student participation in the design and planning of whole‐school interventions. Student participation denotes the involvement of students in decision‐making concerning the design, planning, implementation and/or evaluation of interventions (Griebler et al. 2017). All included studies involved students in the evaluation process, while 61% (17 studies) also included students in the implementation of intervention strategies. In relation to the latter, students occasionally assumed an active role in not only implementing, but also planning intervention strategies, through serving as members of school action teams (6 studies), developing and implementing whole‐school activities (4 studies), and co‐developing school policies (4 studies). However, in 10 studies, students simply implemented prescribed intervention strategies. Less than one‐third of studies referenced student participation in the design of whole‐school interventions. A lack of student involvement in the design and planning of whole‐school interventions, however, raises the possibility of a mismatch between interventions and the real‐world needs, preferences and interests of adolescents. For example, Sawyer, Pfeiffer, et al. (2010) concluded that while their intervention was based on the best evidence, the programme was evaluated as ineffective and feedback from adolescents indicated that only 30% would recommend it to a friend (Sawyer, Pfeiffer, et al. 2010). While there is a paucity of qualitative research concerning whole‐school interventions in school‐aged populations (Langford et al. 2015), existing research raises concern regarding this mismatch. For example, while our review identified posters to be the most common strategy employed in the physical environment domain, qualitative research with adolescents instead raised their desire for safe spaces in schools (Spencer et al. 2022); a strategy not reported by any of the included studies. Beyond ensuring that interventions reflect the needs, preferences and interests of adolescents, a systematic review on the effects of student participation in school health promotion highlights a variety of other benefits (Griebler et al. 2017). These include greater student satisfaction, motivation and ownership of interventions; improved health literacy and health behaviours among students; and improved interactions with peers and in adult‐student relationships. The review also identified two major negative consequences associated with student participation, where in certain studies, students felt dismissed or not taken seriously, or found their participation to be too challenging and interfering with their schoolwork. This cautions against the tokenistic involvement of students and the need for students to be appropriately supported throughout the process of participation. In involving students in the design and planning of interventions, it is crucial that there is adequate representation of students from marginalised backgrounds (Cefai et al. 2021) to ensure that whole‐school interventions are inclusive, equitable and sensitive to the diverse needs of the schooling community.

### 6.2 Areas of Major Gaps in the Evidence

Reflecting on the target population of the included studies, we highlight several key considerations for researchers, public health and policy makers. Consistent with findings from a prior systematic review (Langford et al. 2015), there was minimal representation of low‐ and middle‐income countries in our EGM (5 of 28 studies). Given that the majority of those with mental illness reside in low‐ and middle‐income settings (Ojagbemi and Gureje 2022), there is a need for greater investment in mental health promotive and preventive efforts in these countries. A preponderance of studies also focused on students in the lower half of secondary school, with comparatively little attention invested in those from grade 10 and beyond (5 of 28 studies). Potential mechanisms underlying this disparity may include the competing responsibilities of schools and overcrowded academic curricula; barriers that are likely to intensify as students enter upper secondary school and complete examinations governing university entrance (Spencer et al. 2022; Nguyen et al. 2013). However, given the higher prevalence of mental illness observed in senior secondary school students as compared to their junior secondary school counterparts (Deng et al. 2023), it is crucial that stakeholders consider how this older adolescent category can continue to be engaged.

Several foci are identified for future research to strengthen whole‐school interventions promoting mental health and preventing risk behaviours in adolescence. The first is the need for studies to provide sufficient information on the implementation of each domain of a whole‐school approach, to promote (i) understanding of the study context; (ii) replication of research; and (iii) implementation of intervention strategies in real‐world settings. Secondly, given the varying frequency with which the eight domains were addressed, we recommend that future research investigates whether certain domains are critical to intervention success and whether addressing more domains translates to greater impact. Thirdly, while our review emphasises the overlap in intervention strategies within each domain, we recommend that future research examines the relative effectiveness of common strategies to enable the most effective to be prioritised by stakeholders such as schools and public health and policy makers.

Finally, we highlight four factors worthwhile consideration in the design and planning of whole‐school interventions. Firstly, we recommend that stakeholders involved in intervention design consider and investigate the impact of initial training (addressed by 82% of included studies) and ongoing support (addressed by 46% of included studies) on implementation quality, given that implementation quality has been identified as a critical ingredient to whole‐school intervention success (Durlak et al. 2011). Secondly, given that multiple risk behaviour interventions were a common intervention type and are more aligned with the theoretical underpinnings of a whole‐school approach, we recommend that future research investigate their comparative effectiveness with single‐issue preventive strategies. Thirdly, in line with a previous systematic review on community‐based health promotion initiatives (Merzel and D'Afflitti 2003), the interventions implemented by studies in our EGM typically spanned 2 to 3 years in duration. As similarly raised by these review authors, we bring into question whether such short durations are adequate in achieving the complex systems‐level change aspired for by whole‐school interventions. Finally, we recommend greater student involvement in the design and planning of whole‐school interventions, to ensure that whole‐school interventions capture their real‐world needs, preferences and interests.

### 6.3 Potential Biases in the Mapping Process

We limited potential bias in the EGM by conducting the review in alignment with a pre‐registered protocol. Potential bias was further limited through implementing a systematic search strategy across eight scientific databases, alongside grey literature sources identified by experts in the subject area. Our review team also included an experienced research librarian who was an information retrieval expert to plan, pilot and conduct all searches.

### 6.4 Limitations of the EGM

In placing our findings in perspective, it is important to consider several limitations. Firstly, we did not include study designs that were not randomised controlled trials. Interventions that are implemented in more naturalistic settings, which may be more conducive to quasi‐experimental designs (Barry et al. 2019), may have thus been missed. For example, a school may implement multiple discrete interventions in achieving a whole‐school approach, such as one intervention to address the curriculum‐level, a second to address the ethos and environment‐level, and a third to address the community‐level. Our decision to include only randomised controlled trials, however, was informed by these representing the most robust trial design for evaluation studies. Secondly, the included studies varied in study quality. While most studies were classified as low or unclear risk for each of the risk‐of‐bias subcategories, detection bias (Domain 4) served as an exception, with 25 of 28 studies classifying as high risk‐of‐bias. This was because many studies did not provide sufficient information on whether participants had been blinded to the trial, in the context of the difficulties encountered in blinding for this intervention type. Thirdly, our review did not examine the effectiveness of the whole‐school interventions implemented by the included studies, nor the relative effectiveness of the intervention strategies mapped against the eight domains. The purpose of this review, however, was to appraise current operationalisation of whole‐school interventions promoting mental health and preventing risk behaviours in adolescent against the recommendations of the Health‐Promoting Schools Framework to identify critical evidence gaps. A meta‐analysis of the effectiveness of the interventions implemented by the included studies is presented in a separate article (Balasooriya Lekamge et al. 2025). A final limitation is that our EGM included studies that fulfilled the basic requirement of including a programme component at each of the curriculum‐, ethos and environment‐, and community‐levels, irrespective of whether the Health‐Promoting Schools Framework informed their design. While this was performed in alignment with previous reviews (Goldberg et al. 2019; Langford et al. 2015), it is worthwhile for future reviews to consider whether studies must explicitly reference the Health‐Promoting Schools Framework as their theoretical basis to ensure that these interventions consider themselves to be whole‐school interventions. Despite these limitations, our findings are derived from a large‐scale systematic EGM including 28 studies reported in 58 publications, and fills an important evidence gap in the literature. The findings will provide stakeholders such as schools, researchers, public health and policy makers with a greater understanding of the current operationalisation of whole‐school interventions promoting mental health and preventing risk behaviours in adolescence and has enabled identification of evidence gaps for future research to support optimal translation of the Health‐Promoting Schools Framework into practice.

## 7 Implications for Research, Practice and/or Policy

Our review provides a synthesis of intervention strategies by the eight domains of a whole‐school approach, affording stakeholders including researchers, schools, public health and policy makers greater clarity on current practice and critical gaps in the evidence. Our findings identify the need for greater investment in evaluation studies in low‐ and middle‐income countries and among older adolescent populations. Future quantitative studies should investigate whether certain domains are critical for intervention success, whether addressing more domains translates to greater impact, and the relative effectiveness of overlapping intervention strategies to enable the most effective to be prioritised. We urge consideration and investigation of the impact of design factors on intervention success, including intervention duration, intervention type (single‐issue vs. multiple‐issue prevention), and the provision of training and support mechanisms. We highlight the need for greater student involvement in the design and planning of whole‐school interventions, to ensure that interventions mirror their needs, preferences and interests, encourage student buy‐in and optimise the likelihood of intervention success.

## Author Contributions

- Conceptualisation: Roshini Balasooriya Lekamge, Leo Chen, Nazmul Karin, Margaret M. Barry, Dragan Ilic.
- EGM methods: Roshini Balasooriya Lekamge, Leo Chen, Nazmul Karin, Margaret M. Barry, Dragan Ilic.
- Information retrieval: Roshini Balasooriya Lekamge, Ria Jain, Jenny Sheen, Pravik Solanki, Yida Zhou, Lorena Romero.
- Formal analysis: Roshini Balasooriya Lekamge, Ria Jain, Jenny Sheen, Pravik Solanki, Yida Zhou.
- EGM write‐up–original draft: Roshini Balasooriya Lekamge.
- EGM write‐up–review and editing: Ria Jain, Jenny Sheen, Pravik Solanki, Yida Zhou, Lorena Romero, Leo Chen, Nazmul Karin, Margaret M. Barry, Dragan Ilic.

## Conflicts of Interest

The authors declare no conflicts of interest.

## Differences Between Protocol and Full Report

This EGM was conducted in accordance with a pre‐registered protocol (PROSPERO ID: CRD42023491619). Two amendments were made to the original protocol (ID: CRD42023457678) during the title and abstract screening phase: (i) the addition of 3 databases (CENTRAL, ERIC, Scopus) and (ii) narrowing of the intervention inclusion criteria to include universal whole‐school interventions (this is a subset of the original inclusion criteria, universal school‐based interventions).

## Supporting information

Supporting information.

Supporting information.

Supporting information.

Supporting information.
