# Supplementary material for: Evidence and Gap Map of Whole‐School Interventions Promoting Mental Health and Preventing Risk Behaviours in Adolescence: Programme Component Mapping Within the Health‐Promoting Schools Framework: An evidence and gap map
Source: Campbell Syst Rev. 2025 Mar 10;21(1):e70024. doi: 10.1002/cl2.70024 (PMC11891928; doi:10.1002/cl2.70024)
Supplement: Supplementary file 2 — Supporting information. [file CL2-21-e70024-s003.docx]

**Characteristics of excluded studies**

| Study | Reason for exclusion |
| --- | --- |
| Buttigieg et al., 2015 | Wrong intervention |
| Gabriel et al., 1996 | Wrong study design |
| Orpinas et al., 2000 | Wrong population |
| Russell-Mayhew et al., 2007 | Wrong study design |
| Toumbourou et al., 2013 | Wrong intervention |
| Busch et al., 2013 | Wrong study design |
| Shisslak et al., 1990 | Wrong study design |

**References**

Busch, V., R. J. J. De Leeuw, and A. J. P. Schrijvers. 2013. “Results of a

Multibehavioral Health‐Promoting School Pilot Intervention in a Dutch

Secondary School.” *Journal of Adolescent Health* 52, no. 4: 400–406.

https://doi.org/10.1016/j.jadohealth.2012.07.008.

Buttigieg, J. P., A. L. Shortt, T. M. Slaviero, D. Hutchinson, P. Kremer,

and J. W. Toumbourou. 2015. “A Longitudinal Evaluation of the

Resilient Families Randomized Trial to Prevent Early Adolescent

Depressive Symptoms.” *Journal of Adolescence* 44, no. 1: 204–213.

<https://doi.org/10.1016/j.adolescence.2015.07.014>.

Gabriel, R. M., T. Hopson, M. Haskins, and K. E. Powell. 1996.

“Building Relationships and Resilience in the Prevention of Youth

Violence.” *American Journal of Preventive Medicine* 12, no. 5: 48–55.

<https://doi.org/10.1016/S0749-3797(18)30236-8>.

Orpinas, P. 2000. “Outcome Evaluation of a Multi‐Component

Violence‐Prevention Program for Middle Schools: The Students for

Peace Project.” *Health Education Research* 15, no. 1: 45–58. https://doi.

org/10.1093/her/15.1.45.

Russell‐Mayhew, S., N. Arthur, and C. Ewashen. 2007. “Targeting

Students, Teachers and Parents in a Wellness‐Based Prevention Program

in Schools.” *Eating Disorders* 15, no. 2: 159–181. https://doi.org/

10.1080/10640260701190709.

Shisslak, C. M., M. Crago, and M. E. Neal. 1990. “Prevention of Eating

Disorders Among Adolescents.” *American Journal of Health Promotion*

5, no. 2: 100–106.

Toumbourou, J. W., M. E. D. Gregg, A. L. Shortt, D. M. Hutchinson, and

T. M. Slaviero. 2013. “Reduction of Adolescent Alcohol Use Through

Family–School Intervention: A Randomized Trial.” *Journal of Adolescent*

*Health* 53, no. 6: 778–784. https://doi.org/10.1016/j.jadohealth.2013.07.005.
