# Supplementary material for: Evidence and Gap Map of Whole‐School Interventions Promoting Mental Health and Preventing Risk Behaviours in Adolescence: Programme Component Mapping Within the Health‐Promoting Schools Framework: An evidence and gap map
Source: Campbell Syst Rev. 2025 Mar 10;21(1):e70024. doi: 10.1002/cl2.70024 (PMC11891928; doi:10.1002/cl2.70024)
Supplement: Supplementary file 4 — Supporting information. [file CL2-21-e70024-s004.docx]

SUPPLEMENTARY MATIERIALS 1:
SEARCH STRATEGY ACROSS ALL DATABASES

**Database: Ovid MEDLINE(R)**

MEDLINE search strategy reproduced from Balasooriya et al. (2025) as published in Springer Nature, under the CC BY license: <https://creativecommons.org/licenses/>.

| **#** | **Searches** |
| --- | --- |
| 1 | curriculum/ or schools/ or School Teachers/ or School Health Services/ |
| 2 | (curriculum or curricula* or ((classroom or class room or schoolbased or school based or school setting* or whole school or school approach or school policies or school policy or school health polic* or school wide or schoolwide or school delivered or school led or teacher led or school teacher* or school climate or school community or teaching staff* or school staff* or core school subject?) adj8 (intervention* or program*))).mp. |
| 3 | ((classroom or class room or schoolbased or school based or school setting* or whole school or school approach or school policies or school policy or school health polic* or school wide or schoolwide or school delivered or school led or teacher led or school teacher* or school climate or school community or school teaching staff* or school staff* or core school subject?) adj (initiative* or project* or strategy)).mp. |
| 4 | ((universal or school or teacher-led or teacherled or student wide or studentwide or student based or studentbased) adj3 (intervention* or program* or initiative* or project* or strategy)).mp. |
| 5 | (((system wide or systemwide or population wide or populationwide or population based or populationbased) adj3 (intervention* or program*)) and (school or highschool)).mp. |
| 6 | (((tier 1 or tier one) adj (intervention* or program*)) and (school or highschool)).mp. |
| 7 | ((embed* or delivered or implemented or integrated or incorporated) adj4 (school? or teacher* or highschool*)).mp. |
| 8 | 1 or 2 or 3 or 4 or 5 or 6 or 7 |
| 9 | Adolescent/ or Students/ |
| 10 | (adolescen* or teen* or secondary school* or school student? or school girl? or school boy? or high school or middle school or grade school).mp. |
| 11 | ((child* or boy? or girl?) adj aged adj3 (12 years or 13 years or 14 years or 15 years or 16 years or 17 years or 18 years or twelve years or thirteen years or fourteen years or fifteen years or sixteen years or seventeen years or eighteen years)).mp. |
| 12 | ((child* or boy? or girl?) adj4 (12 year? Old? or 13 year? Old? or 14 year? Old? or 15 year? Old? or 16 year? Old? or 17 year? Old? or 18 year? Old? or 12 years of age or 13 years of age or 14 years of age or 15 years of age or 16 years of age or 17 years of age or 18 years of age)).mp. |
| 13 | ((child* or boy? or girl?) adj3 (age* twelve or age* thirteen or age* fourteen or age* fifteen or age* sixteen or age* seventeen or age* eighteen or age* 12 or age* 13 or age* 14 or age* 15 or age* 16 or age* 17 or age* 18 or age* of twelve or age* of thirteen or age* of fourteen or age* of fifteen or age* of sixteen or age* of seventeen or age* of eighteen or age* of 12 or age* of 13 or age* of 14 or age* of 15 or age* of 16 or age* of 17 or age* of 18)).mp. |
| 14 | ((child* or boy? or girl?) adj4 (grade 7 or grade 8 or grade 9 or grade 10 or grade 11 or grade 12 or year 7 or year 8 or year 9 or year 10 or year 11 or year 12)).mp. |
| 15 | 9 or 10 or 11 or 12 or 13 or 14 |
| 16 | Mental health/ or Emotions/ or Psychological wellbeing/ or Personal satisfaction/ or mindfulness/ or resilience, psychological/ or happiness/ or hope/ or Emotional regulation/ or affect/ or interpersonal relations/ or quality of life/ or Social integration/ or Social cohesion/ or Social adjustment/ or Social responsibility/ or Happiness/ or Social skills/ or Social identification/ or Self-concept/ or Self-efficacy/ or Sense of coherence/ |
| 17 | (mental health or psychological health or stress or emotion* regulation* or emotional health or personal satisfaction or quality of life or interpersonal relation* or social integration or social cohesion or social adjustment or social responsibility or happiness or social skill* or social identification or self-concept or self-efficacy or sense of coherence or social wellbeing or social well-being or life satisfaction or self-esteem or self-confidence or self-belief* or self-advocacy or sense of self or self-awareness or self-acceptance or self-control or self-regulation or self-compassion or coping skill* or problem solving skill* or social connectedness or social belonging or mindfulness).mp. |
| 18 | ((wellness or wellbeing or well-being or empowerment or resilience or resilient or flourishing or thriving or coping) adj5 (psychological* or mental* or emotional*)).mp. |
| 19 | mental disorders/ or anxiety disorders/ or obsessive-compulsive disorder/ or panic disorder/ or phobic disorders/ or phobia, social/ or "disruptive, impulse control, and conduct disorders"/ or dissociative disorders/ or dissociative identity disorder/ or "feeding and eating disorders"/ or anorexia nervosa/ or avoidant restrictive food intake disorder/ or binge-eating disorder/ or bulimia nervosa/ or diabulimia/ or "feeding and eating disorders of childhood"/ or mood disorders/ or "bipolar and related disorders"/ or depressive disorder/ or cyclothymic disorder/ or attention deficit disorder with hyperactivity/ or conduct disorder/ or child behavior disorders/ or reactive attachment disorder/ or schizophrenia, childhood/ or personality disorders/ or antisocial personality disorder/ or borderline personality disorder/ or compulsive personality disorder/ or dependent personality disorder/ or histrionic personality disorder/ or paranoid personality disorder/ or passive-aggressive personality disorder/ or schizoid personality disorder/ or schizotypal personality disorder/ or "schizophrenia spectrum and other psychotic disorders"/ or affective disorders, psychotic/ or psychotic disorders/ or psychoses, substance-induced/ or schizophrenia/ or schizophrenia, catatonic/ or schizophrenia, disorganized/ or schizophrenia, paranoid/ or schizophrenia, treatment-resistant/ or somatoform disorders/ or body dysmorphic disorders/ or body integrity identity disorder/ or conversion disorder/ or factitious disorders/ or munchausen syndrome/ or munchausen syndrome by proxy/ or substance-related disorders/ or alcohol-related disorders/ or alcoholic intoxication/ or alcoholism/ or binge drinking/ or psychoses, alcoholic/ or amphetamine-related disorders/ or cocaine-related disorders/ or inhalant abuse/ or marijuana abuse/ or "marijuana use"/ or narcotic-related disorders/ or neonatal abstinence syndrome/ or phencyclidine abuse/ or substance abuse, intravenous/ or substance abuse, oral/ or "trauma and stressor related disorders"/ or adjustment disorders/ or stress disorders, traumatic/ or stress disorders, post-traumatic/ or stress, psychological/ or burnout, psychological/ or adolescent behavior/ or underage drinking/ or behavioral symptoms/ or affective symptoms/ or delusions/ or depersonalization/ or depression/ or obsessive behavior/ or paranoid behavior/ or problem behavior/ or self-injurious behavior/ or self mutilation/ or suicide/ or suicidal ideation/ or suicide, attempted/ or suicide, completed/ or impulsive behavior/ or compulsive behavior/ or "marijuana use"/ or marijuana smoking/ or "recreational drug use"/ or risk-taking/ or bullying/ or cyberbullying/ |
| 20 | ((mental or anxiety or depression or neurotic or obsessive-compulsive or compulsive-obsessional or panic or phobic or bipolar or disruptive or impulse control or conduct or dissociative or identity or eating or mood or depressive or dysthymic or affective or attention deficit or behavio* or hyperactivity or autism spectrum or autistic or paraphilic or personality or psychotic or paranoid or somatoform or somati#ation or somatic symptom or psychophysiologic* or psychosomatic or body dysmorphic or body integrity identity or stressor related or adjustment or traumatic stress or post-traumatic or posttraumatic or acute traumatic or emotional or disruptive mood dysregulation or substance induced or medication induced or alcohol induced or substance related or alcohol related or drug induced or hoarding or attachment or conversion or bodily distress or functional movement or functional neurological or functional neurologic symptom or sleep wake or sleep initiation or sleep maintenance or insomnia or oppositional defiant or intermittent explosive or factitious or psychoneurotic or manic or cyclothymic or autophag* or schizoaffective or unipolar or delusional or addiction or abnormal psychology) adj disorder*).mp. |
| 21 | (mental illness or psychological illness or psychological distress or mental distress or psychological stress or self-harm or risk taking or risk behavio?r* or suicide or suicidal or bullying or cyberbullying or aggressive behavio?r* or disruptive behavio?r* or behavio?ral problem*).mp. |
| 22 | ((drug* or substanc* or narcotic* or chemical* or heroin* or methamphetamin* or amphetamin* or cocain* or crack* or ice* or tobacco* or smok* or cigarett* or nicotin* or alcohol* or "crystal meth*" or speed* or benzodiazepin* or cannabis* or marijuana*) adj (abuse* or addict* or misuse* or dependen*)).mp. |
| 23 | 16 or 17 or 18 or 19 or 20 or 21 or 22 |
| 24 | (randomized controlled trial or controlled clinical trial).pt. |
| 25 | (randomi?ed or placebo or randomly).ab. or trial.ti. |
| 26 | clinical trials as topic.sh. |
| 27 | 24 or 25 or 26 |
| 28 | 8 and 15 and 23 and 27 |
| 29 | limit 28 to english language |
| 30 | limit 29 to (case reports or comment or editorial or letter or news or newspaper article) |
| 31 | 29 not 30 |
| 32 | 31 not (case report* or comment* or editorial or letter or news*).ti. |
| 33 | limit 32 to ("review" or "systematic review") |
| 34 | 32 not 33 |
| 35 | 34 not review*.ti. |

**Database: Embase**

| **#** | **Searches** |
| --- | --- |
| 1 | curriculum/ or high school/ or middle school/ or school teacher/ or school health service/ |
| 2 | (curriculum or curricula* or ((classroom or class room or schoolbased or school based or school setting* or whole school or school approach or school policies or school policy or school health polic* or school wide or schoolwide or school delivered or school led or teacher led or school teacher* or school climate or school community or teaching staff* or school staff* or core school subject?) adj8 (intervention* or program*))).mp. |
| 3 | ((classroom or class room or schoolbased or school based or school setting* or whole school or school approach or school policies or school policy or school health polic* or school wide or schoolwide or school delivered or school led or teacher led or school teacher* or school climate or school community or school teaching staff* or school staff* or core school subject?) adj (initiative* or project* or strategy)).mp. |
| 4 | ((universal or school or teacher-led or teacherled or student wide or studentwide or student based or studentbased) adj3 (intervention* or program* or initiative* or project* or strategy)).mp. |
| 5 | (((system wide or systemwide or population wide or populationwide or population based or populationbased) adj3 (intervention* or program*)) and (school or highschool)).mp. |
| 6 | (((tier 1 or tier one) adj (intervention* or program*)) and (school or highschool)).mp. |
| 7 | ((embed* or delivered or implemented or integrated or incorporated) adj4 (school? or teacher* or highschool*)).mp. |
| 8 | 1 or 2 or 3 or 4 or 5 or 6 or 7 |
| 9 | adolescent/ or high school student/ |
| 10 | (adolescen* or teen* or secondary school* or school student? or school girl? or school boy? or high school or middle school or grade school).mp. |
| 11 | ((child* or boy? or girl?) adj aged adj3 (12 years or 13 years or 14 years or 15 years or 16 years or 17 years or 18 years or twelve years or thirteen years or fourteen years or fifteen years or sixteen years or seventeen years or eighteen years)).mp. |
| 12 | ((child* or boy? or girl?) adj4 (12 year? Old? or 13 year? Old? or 14 year? Old? or 15 year? Old? or 16 year? Old? or 17 year? Old? or 18 year? Old? or 12 years of age or 13 years of age or 14 years of age or 15 years of age or 16 years of age or 17 years of age or 18 years of age)).mp. |
| 13 | ((child* or boy? or girl?) adj3 (age* twelve or age* thirteen or age* fourteen or age* fifteen or age* sixteen or age* seventeen or age* eighteen or age* 12 or age* 13 or age* 14 or age* 15 or age* 16 or age* 17 or age* 18 or age* of twelve or age* of thirteen or age* of fourteen or age* of fifteen or age* of sixteen or age* of seventeen or age* of eighteen or age* of 12 or age* of 13 or age* of 14 or age* of 15 or age* of 16 or age* of 17 or age* of 18)).mp. |
| 14 | ((child* or boy? or girl?) adj4 (grade 7 or grade 8 or grade 9 or grade 10 or grade 11 or grade 12 or year 7 or year 8 or year 9 or year 10 or year 11 or year 12)).mp. |
| 15 | 9 or 10 or 11 or 12 or 13 or 14 |
| 16 | mental health/ or psychological well-being/ or emotion/ or affect/ or emotion regulation/ or happiness/ or hope/ or satisfaction/ or life satisfaction/ or mindfulness/ or psychological resilience/ or human relation/ or "quality of life"/ or social connectedness/ or social cohesion/ or social adaptation/ or social responsibility/ or social competence/ or social identity/ or self concept/ or self control/ or "sense of coherence"/ or "sense of self"/ |
| 17 | (mental health or psychological health or stress or emotion* regulation* or emotional health or personal satisfaction or quality of life or interpersonal relation* or social integration or social cohesion or social adjustment or social responsibility or happiness or social skill* or social identification or self-concept or self-efficacy or sense of coherence or social wellbeing or social well-being or life satisfaction or self-esteem or self-confidence or self-belief* or self-advocacy or sense of self or self-awareness or self-acceptance or self-control or self-regulation or self-compassion or coping skill* or problem solving skill* or social connectedness or social belonging or mindfulness).mp. |
| 18 | ((wellness or wellbeing or well-being or empowerment or resilience or resilient or flourishing or thriving or coping) adj5 (psychological* or mental* or emotional*)).mp. |
| 19 | mental disease/ or mental instability/ or anxiety disorder/ or acute stress disorder/ or anxiety neurosis/ or generalized anxiety disorder/ or neurosis/ or affective neurosis/ or obsessive compulsive disorder/ or phobia/ or bipolar disorder/ or impulse control disorder/ or behavior disorder/ or attention deficit hyperactivity disorder/ or externalizing disorder/ or oppositional defiant disorder/ or suicidal behavior/ or conduct disorder/ or dissociative disorder/ or depersonalization/ or multiple personality/ or eating disorder/ or anorexia nervosa/ or binge eating disorder/ or bulimia.mp. or mood disorder/ or affective neurosis/ or affective psychosis/ or major affective disorder/ or minor affective disorder/ or schizoaffective psychosis/ or depression/ or agitated depression/ or atypical depression/ or chronic depression/ or depressive psychosis/ or dysphoria/ or dysthymia/ or endogenous depression/ or major depression/ or melancholia/ or minor depression/ or premenstrual dysphoric disorder/ or reactive depression/ or seasonal affective disorder/ or treatment resistant depression/ or personality disorder/ or antisocial personality disorder/ or avoidant personality disorder/ or compulsive personality disorder/ or dependent personality disorder/ or histrionic personality disorder/ or paranoid personality disorder/ or passive aggressive personality disorder/ or schizoidism/ or schizotypal personality disorder/ or delusional disorder/ or psychosis/ or acute psychosis/ or alcohol psychosis/ or brief psychotic disorder/ or drug induced psychosis/ or endogenous psychosis/ or manic psychosis/ or menstrual psychosis/ or paranoid psychosis/ or schizophrenia/ or catatonic schizophrenia/ or paranoid schizophrenia/ or treatment-resistant schizophrenia/ or somatoform disorder/ or body dysmorphic disorder/ or conversion disorder/ or somatic delusion/ or somatization/ or posttraumatic stress disorder/ or adjustment disorder/ or psychotrauma/ or social phobia/ or psychosomatic disorder/ or factitious disease/ or alcoholism/ or amphetamine dependence/ or benzodiazepine dependence/ or cannabis addiction/ or cocaine dependence/ or drug misuse/ or methamphetamine dependence/ or multiple drug abuse/ or narcotic dependence/ or phencyclidine dependence/ or drug abuse/ or alcohol abuse/ or amphetamine abuse/ or analgesic agent abuse/ or illicit drug inhalation/ or inhalant abuse/ or intravenous drug abuse/ or multiple drug abuse/ or phencyclidine abuse/ or prescription drug misuse/ or heroin dependence/ or morphine addiction/ or opiate addiction/ or substance abuse/ or automutilation/ or suicidal behavior/ or self immolation/ or self poisoning/ or suicidal ideation/ or suicide/ or suicide attempt/ or "cannabis use"/ or "substance use"/ or cannabis smoking/ or "recreational drug use"/ or high risk behavior/ or bullying/ or cyberbullying/ |
| 20 | ((mental or anxiety or depression or neurotic or obsessive-compulsive or compulsive-obsessional or panic or phobic or bipolar or disruptive or impulse control or conduct or dissociative or identity or eating or mood or depressive or dysthymic or affective or attention deficit or behavio* or hyperactivity or autism spectrum or autistic or paraphilic or personality or psychotic or paranoid or somatoform or somati#ation or somatic symptom or psychophysiologic* or psychosomatic or body dysmorphic or body integrity identity or stressor related or adjustment or traumatic stress or post-traumatic or posttraumatic or acute traumatic or emotional or disruptive mood dysregulation or substance induced or medication induced or alcohol induced or substance related or alcohol related or drug induced or hoarding or attachment or conversion or bodily distress or functional movement or functional neurological or functional neurologic symptom or sleep wake or sleep initiation or sleep maintenance or insomnia or oppositional defiant or intermittent explosive or factitious or psychoneurotic or manic or cyclothymic or autophag* or schizoaffective or unipolar or delusional or addiction or abnormal psychology) adj disorder*).mp. |
| 21 | (mental illness or psychological illness or psychological distress or mental distress or psychological stress or self-harm or risk taking or risk behavio?r* or suicide or suicidal or bullying or cyberbullying or aggressive behavio?r* or disruptive behavio?r* or behavio?ral problem*).mp. |
| 22 | ((drug* or substanc* or narcotic* or chemical* or heroin* or methamphetamin* or amphetamin* or cocain* or crack* or ice* or tobacco* or smok* or cigarett* or nicotin* or alcohol* or "crystal meth*" or speed* or benzodiazepin* or cannabis* or marijuana*) adj (abuse* or addict* or misuse* or dependen*)).mp. |
| 23 | 16 or 17 or 18 or 19 or 20 or 21 or 22 |
| 24 | clinical trial/ or randomized controlled trial/ or randomization/ or single blind procedure/ or double blind procedure/ or crossover procedure/ or placebo/ or prospective study/ |
| 25 | (randomi?ed controlled or RCT or randomly allocated or allocated randomly or random allocation or (allocated adj2 random) or (single adj1 blind*) or (double adj1 blind*) or ((treble or triple) adj1 blind*) or placebo*).mp. |
| 26 | 24 or 25 |
| 27 | 8 and 15 and 23 and 26 |
| 28 | limit 27 to english language |
| 29 | limit 28 to (conference abstract or editorial or letter or "review") |
| 30 | 28 not 29 |
| 31 | 30 not (case report* or comment* or editorial or letter or news*).ti. |
| 32 | limit 31 to "systematic review" |
| 33 | 31 not 32 |
| 34 | 33 not review*.ti. |

**Database:** **Ovid Emcare**

| **#** | **Searches** |
| --- | --- |
| 1 | curriculum/ or high school/ or middle school/ or school teacher/ or school health service/ |
| 2 | (curriculum or curricula* or ((classroom or class room or schoolbased or school based or school setting* or whole school or school approach or school policies or school policy or school health polic* or school wide or schoolwide or school delivered or school led or teacher led or school teacher* or school climate or school community or teaching staff* or school staff* or core school subject?) adj8 (intervention* or program*))).mp. |
| 3 | ((classroom or class room or schoolbased or school based or school setting* or whole school or school approach or school policies or school policy or school health polic* or school wide or schoolwide or school delivered or school led or teacher led or school teacher* or school climate or school community or school teaching staff* or school staff* or core school subject?) adj (initiative* or project* or strategy)).mp. |
| 4 | ((universal or school or teacher-led or teacherled or student wide or studentwide or student based or studentbased) adj3 (intervention* or program* or initiative* or project* or strategy)).mp. |
| 5 | (((system wide or systemwide or population wide or populationwide or population based or populationbased) adj3 (intervention* or program*)) and (school or highschool)).mp. |
| 6 | (((tier 1 or tier one) adj (intervention* or program*)) and (school or highschool)).mp. |
| 7 | ((embed* or delivered or implemented or integrated or incorporated) adj4 (school? or teacher* or highschool*)).mp. |
| 8 | 1 or 2 or 3 or 4 or 5 or 6 or 7 |
| 9 | adolescent/ or high school student/ |
| 10 | (adolescen* or teen* or secondary school* or school student? or school girl? or school boy? or high school or middle school or grade school).mp. |
| 11 | ((child* or boy? or girl?) adj aged adj3 (12 years or 13 years or 14 years or 15 years or 16 years or 17 years or 18 years or twelve years or thirteen years or fourteen years or fifteen years or sixteen years or seventeen years or eighteen years)).mp. |
| 12 | ((child* or boy? or girl?) adj4 (12 year? Old? or 13 year? Old? or 14 year? Old? or 15 year? Old? or 16 year? Old? or 17 year? Old? or 18 year? Old? or 12 years of age or 13 years of age or 14 years of age or 15 years of age or 16 years of age or 17 years of age or 18 years of age)).mp. |
| 13 | ((child* or boy? or girl?) adj3 (age* twelve or age* thirteen or age* fourteen or age* fifteen or age* sixteen or age* seventeen or age* eighteen or age* 12 or age* 13 or age* 14 or age* 15 or age* 16 or age* 17 or age* 18 or age* of twelve or age* of thirteen or age* of fourteen or age* of fifteen or age* of sixteen or age* of seventeen or age* of eighteen or age* of 12 or age* of 13 or age* of 14 or age* of 15 or age* of 16 or age* of 17 or age* of 18)).mp. |
| 14 | ((child* or boy? or girl?) adj4 (grade 7 or grade 8 or grade 9 or grade 10 or grade 11 or grade 12 or year 7 or year 8 or year 9 or year 10 or year 11 or year 12)).mp. |
| 15 | 9 or 10 or 11 or 12 or 13 or 14 |
| 16 | mental health/ or psychological well-being/ or emotion/ or affect/ or emotion regulation/ or happiness/ or hope/ or satisfaction/ or life satisfaction/ or mindfulness/ or psychological resilience/ or human relation/ or "quality of life"/ or social connectedness/ or social cohesion/ or social adaptation/ or social responsibility/ or social competence/ or social identity/ or self concept/ or self control/ or "sense of coherence"/ or "sense of self"/ |
| 17 | (mental health or psychological health or stress or emotion* regulation* or emotional health or personal satisfaction or quality of life or interpersonal relation* or social integration or social cohesion or social adjustment or social responsibility or happiness or social skill* or social identification or self-concept or self-efficacy or sense of coherence or social wellbeing or social well-being or life satisfaction or self-esteem or self-confidence or self-belief* or self-advocacy or sense of self or self-awareness or self-acceptance or self-control or self-regulation or self-compassion or coping skill* or problem solving skill* or social connectedness or social belonging or mindfulness).mp. |
| 18 | ((wellness or wellbeing or well-being or empowerment or resilience or resilient or flourishing or thriving or coping) adj5 (psychological* or mental* or emotional*)).mp. |
| 19 | mental disease/ or mental instability/ or anxiety disorder/ or acute stress disorder/ or anxiety neurosis/ or generalized anxiety disorder/ or neurosis/ or affective neurosis/ or obsessive compulsive disorder/ or phobia/ or bipolar disorder/ or impulse control disorder/ or behavior disorder/ or attention deficit hyperactivity disorder/ or externalizing disorder/ or oppositional defiant disorder/ or suicidal behavior/ or conduct disorder/ or dissociative disorder/ or depersonalization/ or multiple personality/ or eating disorder/ or anorexia nervosa/ or binge eating disorder/ or bulimia.mp. or mood disorder/ or affective neurosis/ or affective psychosis/ or major affective disorder/ or minor affective disorder/ or schizoaffective psychosis/ or depression/ or agitated depression/ or atypical depression/ or chronic depression/ or depressive psychosis/ or dysphoria/ or dysthymia/ or endogenous depression/ or major depression/ or melancholia/ or minor depression/ or premenstrual dysphoric disorder/ or reactive depression/ or seasonal affective disorder/ or treatment resistant depression/ or personality disorder/ or antisocial personality disorder/ or avoidant personality disorder/ or compulsive personality disorder/ or dependent personality disorder/ or histrionic personality disorder/ or paranoid personality disorder/ or passive aggressive personality disorder/ or schizoidism/ or schizotypal personality disorder/ or delusional disorder/ or psychosis/ or acute psychosis/ or alcohol psychosis/ or brief psychotic disorder/ or drug induced psychosis/ or endogenous psychosis/ or manic psychosis/ or menstrual psychosis/ or paranoid psychosis/ or schizophrenia/ or catatonic schizophrenia/ or paranoid schizophrenia/ or treatment-resistant schizophrenia/ or somatoform disorder/ or body dysmorphic disorder/ or conversion disorder/ or somatic delusion/ or somatization/ or posttraumatic stress disorder/ or adjustment disorder/ or psychotrauma/ or social phobia/ or psychosomatic disorder/ or factitious disease/ or alcoholism/ or amphetamine dependence/ or benzodiazepine dependence/ or cannabis addiction/ or cocaine dependence/ or drug misuse/ or methamphetamine dependence/ or multiple drug abuse/ or narcotic dependence/ or phencyclidine dependence/ or drug abuse/ or alcohol abuse/ or amphetamine abuse/ or analgesic agent abuse/ or illicit drug inhalation/ or inhalant abuse/ or intravenous drug abuse/ or multiple drug abuse/ or phencyclidine abuse/ or prescription drug misuse/ or heroin dependence/ or morphine addiction/ or opiate addiction/ or substance abuse/ or automutilation/ or suicidal behavior/ or self immolation/ or self poisoning/ or suicidal ideation/ or suicide/ or suicide attempt/ or "cannabis use"/ or "substance use"/ or cannabis smoking/ or "recreational drug use"/ or high risk behavior/ or bullying/ or cyberbullying/ |
| 20 | ((mental or anxiety or depression or neurotic or obsessive-compulsive or compulsive-obsessional or panic or phobic or bipolar or disruptive or impulse control or conduct or dissociative or identity or eating or mood or depressive or dysthymic or affective or attention deficit or behavio* or hyperactivity or autism spectrum or autistic or paraphilic or personality or psychotic or paranoid or somatoform or somati#ation or somatic symptom or psychophysiologic* or psychosomatic or body dysmorphic or body integrity identity or stressor related or adjustment or traumatic stress or post-traumatic or posttraumatic or acute traumatic or emotional or disruptive mood dysregulation or substance induced or medication induced or alcohol induced or substance related or alcohol related or drug induced or hoarding or attachment or conversion or bodily distress or functional movement or functional neurological or functional neurologic symptom or sleep wake or sleep initiation or sleep maintenance or insomnia or oppositional defiant or intermittent explosive or factitious or psychoneurotic or manic or cyclothymic or autophag* or schizoaffective or unipolar or delusional or addiction or abnormal psychology) adj disorder*).mp. |
| 21 | (mental illness or psychological illness or psychological distress or mental distress or psychological stress or self-harm or risk taking or risk behavio?r* or suicide or suicidal or bullying or cyberbullying or aggressive behavio?r* or disruptive behavio?r* or behavio?ral problem*).mp. |
| 22 | ((drug* or substanc* or narcotic* or chemical* or heroin* or methamphetamin* or amphetamin* or cocain* or crack* or ice* or tobacco* or smok* or cigarett* or nicotin* or alcohol* or "crystal meth*" or speed* or benzodiazepin* or cannabis* or marijuana*) adj (abuse* or addict* or misuse* or dependen*)).mp. |
| 23 | 16 or 17 or 18 or 19 or 20 or 21 or 22 |
| 24 | clinical trial/ or randomized controlled trial/ or randomization/ or single blind procedure/ or double blind procedure/ or crossover procedure/ or placebo/ or prospective study/ |
| 25 | (randomi?ed controlled or RCT or randomly allocated or allocated randomly or random allocation or (allocated adj2 random) or (single adj1 blind*) or (double adj1 blind*) or ((treble or triple) adj1 blind*) or placebo*).mp. |
| 26 | 24 or 25 |
| 27 | 8 and 15 and 23 and 26 |
| 28 | limit 27 to english language |
| 29 | limit 28 to (conference abstract or editorial or letter or "review") |
| 30 | 28 not 29 |
| 31 | 30 not (case report* or comment* or editorial or letter or news*).ti. |
| 32 | limit 31 to "systematic review" |
| 33 | 31 not 32 |
| 34 | 33 not review*.ti. |

**Database: APA PsycInfo**

| **#** | **Searches** |
| --- | --- |
| 1 | curriculum/ or high schools/ or high school education/ or secondary education/ or high school teachers/ or junior high school teachers/ or middle school teachers/ or school health service/ |
| 2 | (curriculum or curricula* or ((classroom or class room or schoolbased or school based or school setting* or whole school or school approach or school policies or school policy or school health polic* or school wide or schoolwide or school delivered or school led or teacher led or school teacher* or school climate or school community or teaching staff* or school staff* or core school subject?) adj8 (intervention* or program*))).mp. |
| 3 | ((classroom or class room or schoolbased or school based or school setting* or whole school or school approach or school policies or school policy or school health polic* or school wide or schoolwide or school delivered or school led or teacher led or school teacher* or school climate or school community or school teaching staff* or school staff* or core school subject?) adj (initiative* or project* or strategy)).mp. |
| 4 | ((universal or school or teacher-led or teacherled or student wide or studentwide or student based or studentbased) adj3 (intervention* or program* or initiative* or project* or strategy)).mp. |
| 5 | (((system wide or systemwide or population wide or populationwide or population based or populationbased) adj3 (intervention* or program*)) and (school or highschool)).mp. |
| 6 | (((tier 1 or tier one) adj (intervention* or program*)) and (school or highschool)).mp. |
| 7 | ((embed* or delivered or implemented or integrated or incorporated) adj4 (school? or teacher* or highschool*)).mp. |
| 8 | 1 or 2 or 3 or 4 or 5 or 6 or 7 |
| 9 | adolescent/ or high school student/ |
| 10 | (adolescen* or teen* or secondary school* or school student? or school girl? or school boy? or high school or middle school or grade school).mp. |
| 11 | ((child* or boy? or girl?) adj aged adj3 (12 years or 13 years or 14 years or 15 years or 16 years or 17 years or 18 years or twelve years or thirteen years or fourteen years or fifteen years or sixteen years or seventeen years or eighteen years)).mp. |
| 12 | ((child* or boy? or girl?) adj4 (12 year? Old? or 13 year? Old? or 14 year? Old? or 15 year? Old? or 16 year? Old? or 17 year? Old? or 18 year? Old? or 12 years of age or 13 years of age or 14 years of age or 15 years of age or 16 years of age or 17 years of age or 18 years of age)).mp. |
| 13 | ((child* or boy? or girl?) adj3 (age* twelve or age* thirteen or age* fourteen or age* fifteen or age* sixteen or age* seventeen or age* eighteen or age* 12 or age* 13 or age* 14 or age* 15 or age* 16 or age* 17 or age* 18 or age* of twelve or age* of thirteen or age* of fourteen or age* of fifteen or age* of sixteen or age* of seventeen or age* of eighteen or age* of 12 or age* of 13 or age* of 14 or age* of 15 or age* of 16 or age* of 17 or age* of 18)).mp. |
| 14 | ((child* or boy? or girl?) adj4 (grade 7 or grade 8 or grade 9 or grade 10 or grade 11 or grade 12 or year 7 or year 8 or year 9 or year 10 or year 11 or year 12)).mp. |
| 15 | 9 or 10 or 11 or 12 or 13 or 14 |
| 16 | mental health/ or emotional health/ or well being/ or interpersonal relationships/ or "resilience (psychological)"/ or emotional regulation/ or emotion/ or happiness/ or hope/ or satisfaction/ or life satisfaction/ or mindfulness/ or "quality of life"/ or social connectedness/ or social cohesion/ or social adaptation/ or social responsibility/ or social competence/ or social identity/ or self concept/ or self control/ or "sense of coherence"/ or "sense of self"/ |
| 17 | (mental health or psychological health or stress or emotion* regulation* or emotional health or personal satisfaction or quality of life or interpersonal relation* or social integration or social cohesion or social adjustment or social responsibility or happiness or social skill* or social identification or self-concept or self-efficacy or sense of coherence or social wellbeing or social well-being or life satisfaction or self-esteem or self-confidence or self-belief* or self-advocacy or sense of self or self-awareness or self-acceptance or self-control or self-regulation or self-compassion or coping skill* or problem solving skill* or social connectedness or social belonging or mindfulness).mp. |
| 18 | ((wellness or wellbeing or well-being or empowerment or resilience or resilient or flourishing or thriving or coping) adj5 (psychological* or mental* or emotional*)).mp. |
| 19 | mental disorders/ or affective disorders/ or anxiety disorders/ or bipolar disorder/ or borderline states/ or chronic mental illness/ or dissociative disorders/ or eating disorders/ or gender dysphoria/ or neurosis/ or paraphilias/ or personality disorders/ or psychosis/ or serious mental illness/ or somatoform disorders/ or "stress and trauma related disorders"/ or "substance related and addictive disorders"/ or disruptive mood dysregulation disorder/ or seasonal affective disorder/ or major depression/ or anaclitic depression/ or dysthymic disorder/ or endogenous depression/ or reactive depression/ or recurrent depression/ or treatment resistant depression/ or generalized anxiety disorder/ or obsessive compulsive disorder/ or panic disorder/ or phobias/ or mania/ or hypomania/ or acute psychosis/ or affective psychosis/ or alcoholic psychosis/ or chronic psychosis/ or hallucinosis/ or "paranoia (psychosis)"/ or reactive psychosis/ or toxic psychoses/ or alcoholic hallucinosis/ or korsakoffs psychosis/ or depersonalization/ or "depersonalization/derealization disorder"/ or dissociative amnesia/ or dissociative identity disorder/ or anorexia nervosa/ or binge eating disorder/ or bulimia/ or feeding disorders/ or "purging (eating disorders)"/ or attention deficit disorder/ or disruptive behavior disorders/ or "emotional and behavioral disorders"/ or attention deficit disorder with hyperactivity/ or conduct disorder/ or oppositional defiant disorder/ or traumatic neurosis/ or schizophrenia/ or acute schizophrenia/ or catatonic schizophrenia/ or paranoid schizophrenia/ or process schizophrenia/ or schizoaffective disorder/ or "schizophrenia (disorganized type)"/ or schizophreniform disorder/ or undifferentiated schizophrenia/ or schizoid personality disorder/ or schizotypal personality disorder/ or schizotypy/ or antisocial personality disorder/ or avoidant personality disorder/ or borderline personality disorder/ or dependent personality disorder/ or histrionic personality disorder/ or narcissistic personality disorder/ or obsessive compulsive personality disorder/ or paranoid personality disorder/ or passive aggressive personality disorder/ or body dysmorphic disorder/ or conversion disorder/ or factitious disorders/ or somatization disorder/ or acute stress disorder/ or adjustment disorders/ or posttraumatic stress disorder/ or complex ptsd/ or addiction/ or "alcohol use disorder"/ or "cannabis use disorder"/ or drug abuse/ or drug dependency/ or inhalant abuse/ or "opioid use disorder"/ or prescription drug misuse/ or polydrug abuse/ or alcoholism/ or binge drinking/ or heroin addiction/ or morphine dependence/ or impulse control disorders/ or explosive disorder/ or psychopathology/ or abnormal psychology/ or self-injurious behavior/ or self-destructive behavior/ or self-inflicted wounds/ or self-mutilation/ or self-poisoning/ or suicide/ or suicidality/ or suicidal ideation/ or attempted suicide/ or "cannabis use"/ or "substance use"/ or cannabis smoking/ or "recreational drug use"/ or high risk behavior/ or bullying/ or cyberbullying/ |
| 20 | ((mental or anxiety or depression or neurotic or obsessive-compulsive or compulsive-obsessional or panic or phobic or bipolar or disruptive or impulse control or conduct or dissociative or identity or eating or mood or depressive or dysthymic or affective or attention deficit or behavio* or hyperactivity or autism spectrum or autistic or paraphilic or personality or psychotic or paranoid or somatoform or somati#ation or somatic symptom or psychophysiologic* or psychosomatic or body dysmorphic or body integrity identity or stressor related or adjustment or traumatic stress or post-traumatic or posttraumatic or acute traumatic or emotional or disruptive mood dysregulation or substance induced or medication induced or alcohol induced or substance related or alcohol related or drug induced or hoarding or attachment or conversion or bodily distress or functional movement or functional neurological or functional neurologic symptom or sleep wake or sleep initiation or sleep maintenance or insomnia or oppositional defiant or intermittent explosive or factitious or psychoneurotic or manic or cyclothymic or autophag* or schizoaffective or unipolar or delusional or addiction or abnormal psychology) adj disorder*).mp. |
| 21 | (mental illness or psychological illness or psychological distress or mental distress or psychological stress or self-harm or risk taking or risk behavio?r* or suicide or suicidal or bullying or cyberbullying or aggressive behavio?r* or disruptive behavio?r* or behavio?ral problem*).mp. |
| 22 | ((drug* or substanc* or narcotic* or chemical* or heroin* or methamphetamin* or amphetamin* or cocain* or crack* or ice* or tobacco* or smok* or cigarett* or nicotin* or alcohol* or "crystal meth*" or speed* or benzodiazepin* or cannabis* or marijuana*) adj (abuse* or addict* or misuse* or dependen*)).mp. |
| 23 | 16 or 17 or 18 or 19 or 20 or 21 or 22 |
| 24 | randomized controlled trials/ or clinical trials/ or randomized clinical trials/ or Random Sampling/ or placebo/ or prospective studies/ |
| 25 | (randomi?ed controlled or RCT or randomly allocated or allocated randomly or random allocation or (allocated adj2 random) or (single adj1 blind*) or (double adj1 blind*) or ((treble or triple) adj1 blind*) or placebo*).mp. |
| 26 | 24 or 25 |
| 27 | 8 and 15 and 23 and 26 |
| 28 | limit 27 to english language |
| 29 | limit 28 to ("column/opinion" or "comment/reply" or dissertation or editorial or encyclopedia entry or letter) |
| 30 | 28 not 29 |
| 31 | limit 30 to (review-book or review-media or review-software & other or reviews) |
| 32 | 30 not 31 |
| 33 | 32 not (case report* or comment* or editorial or letter or news* or dissertation).ti. |
| 34 | 33 not review*.ti. |

**Database: CINAHL**

| **#** | **Query** |
| --- | --- |
| S34 | S31 NOT S32  Limiters - English Language |
| S33 | S31 NOT S32 |
| S32 | TI ( (Anecdote OR Brief Item OR Commentary OR Dissertation OR Editorial OR "Legal Case" OR Letter OR Thesis OR Pamphlet OR Review OR "Systematic Review") ) OR TI ( "case report*" OR news* or magazine* ) |
| S31 | S29 NOT S30 |
| S30 | PT (Anecdote OR Book Review OR Brief Item OR Commentary OR Doctoral Dissertation OR Editorial OR Legal Case OR Letter OR Masters Thesis OR Pamphlet OR Review OR Systematic Review OR Website) |
| S29 | S8 AND S15 AND S23 AND S28 |
| S28 | S24 OR S25 OR S26 OR S27 |
| S27 | (MH "Quantitative Studies") OR (MH "Placebos") |
| S26 | TX ( (singl* N1 blind*) or (singl* N1 mask*) ) or TX ( (doubl* N1 blind*) or (doubl* N1 mask*) ) or TX ( (tripl* N1 blind*) or (tripl* N1 mask*) ) or TX ( (trebl* N1 blind*) or (trebl* N1 mask*) ) |
| S25 | (MH "Random Assignment") OR TX "randomi* control* trial*" OR TX "random* allocat*" OR TX "allocat* random*" |
| S24 | (MH "Clinical Trials+") OR PT Clinical trial OR TX (clinical* N1 trial*) |
| S23 | S16 OR S17 OR S18 OR S19 OR S20 OR S21 OR S22 |
| S22 | ((drug* or substanc* or narcotic* or chemical* or heroin* or methamphetamin* or amphetamin* or cocain* or crack* or ice* or tobacco* or smok* or cigarett* or nicotin* or alcohol* or "crystal meth*" or speed* or benzodiazepin* or cannabis* or marijuana*) N0 (abuse* or addict* or misuse* or dependen*)) |
| S21 | (“mental illness” or “psychological illness” or “psychological distress” or “mental distress” or “psychological stress” or “self-harm” or “risk taking” or “risk behavio?r*” or suicide or suicidal or bullying or cyberbullying or “aggressive behavio?r*” or “disruptive behavio?r*” or “behavio?ral problem*”) |
| S20 | ((mental or anxiety or depression or neurotic or “obsessive-compulsive” or “compulsive-obsessional” or panic or phobic or bipolar or disruptive or “impulse control” or conduct or dissociative or identity or eating or mood or depressive or dysthymic or affective or “attention deficit” or behavio* or hyperactivity or “autism spectrum” or autistic or paraphilic or personality or psychotic or paranoid or somatoform or somati#ation or “somatic symptom” or psychophysiologic* or psychosomatic or “body dysmorphic” or “body integrity identity” or “stressor related” or adjustment or “traumatic stress” or “post-traumatic” or posttraumatic or “acute traumatic” or emotional or “disruptive mood dysregulation” or “substance induced” or “medication induced” or “alcohol induced” or “substance related” or “alcohol related” or “drug induced” or hoarding or attachment or conversion or “bodily distress” or “functional movement” or “functional neurological” or “functional neurologic symptom” or “sleep wake” or “sleep initiation” or “sleep maintenance” or insomnia or “oppositional defiant” or “intermittent explosive” or factitious or psychoneurotic or manic or cyclothymic or autophag* or schizoaffective or unipolar or delusional or addiction or “abnormal psychology”) N0 disorder*) |
| S19 | ( (MH "Mental Disorders") OR (MH "Adjustment Disorders") OR (MH "Depression, Reactive") OR (MH "Mental Disorders, Chronic") OR (MH "Attention Deficit Hyperactivity Disorder") OR (MH "Neurotic Disorders") OR (MH "Affective Disorders") OR (MH "Depression") OR (MH "Dysthymic Disorder") OR (MH "Seasonal Affective Disorder") OR (MH "Premenstrual Dysphoric Disorder") OR (MH "Anxiety Disorders") OR (MH "Generalized Anxiety Disorder") OR (MH "Obsessive-Compulsive Disorder") OR (MH "Body Dysmorphic Disorder") OR (MH "Panic Disorder") OR (MH "Phobic Disorders") OR (MH "Social Anxiety Disorders") OR (MH "Stress Disorders, Post-Traumatic") OR (MH "Factitious Disorders") OR (MH "Body Integrity Identity Disorder") OR (MH "Somatoform Disorders") OR (MH "Dissociative Disorders") OR (MH "Multiple-Personality Disorder") OR (MH "Organic Mental Disorders") OR (MH "Organic Mental Disorders, Psychotic") OR (MH "Psychoses, Substance-Induced") OR (MH "Psychoses, Alcoholic") OR (MH "Organic Mental Disorders, Substance-Induced") OR (MH "Paraphilias") OR (MH "Personality Disorders") OR (MH "Antisocial Personality Disorder") OR (MH "Avoidant Personality Disorder") OR (MH "Borderline Personality Disorder") OR (MH "Compulsive Personality Disorder") OR (MH "Dependent Personality Disorder") OR (MH "Diogenes Syndrome") OR (MH "Histrionic Personality Disorder") OR (MH "Impulse Control Disorders") OR (MH "Narcissistic Personality Disorder") OR (MH "Passive-Aggressive Personality Disorder") OR (MH "Schizotypal Personality Disorder") OR (MH "Psychotic Disorders") OR (MH "Affective Disorders, Psychotic") OR (MH "Bipolar Disorder") OR (MH "Cyclothymic Disorder") OR (MH "Paranoid Disorders") OR (MH "Schizoaffective Disorder") OR (MH "Schizophrenia") OR (MH "Schizophrenia, Treatment-Resistant") OR (MH "Substance Use Disorders") OR (MH "Substance Abuse") OR (MH "Alcohol-Related Disorders") OR (MH "Alcoholism") OR (MH "Inhalant Abuse") OR (MH "Substance Abuse, Intravenous") OR (MH "Psychological Trauma") OR (MH "Eating Disorders") OR (MH "Anorexia") OR (MH "Anorexia Nervosa") OR (MH "Binge Eating Disorder") OR (MH "Avoidant Restrictive Food Intake Disorder") OR (MH "Bulimia") OR (MH "Bulimia Nervosa") OR (MH "Orthorexia Nervosa") OR ( (MH "Suicide") OR (MH "Suicidal Ideation") OR (MH "Suicide, Attempted") OR (MH "Injuries, Self-Inflicted") OR (MH "Self-Injurious Behavior") OR (MH "Psychophysiologic Disorders")) OR (MH "Cyberbullying") OR (MH "Bullying") |
| S18 | ((wellness or wellbeing or "well-being" or empowerment or resilience or resilient or flourishing or thriving or coping) N4 (psychological* or mental* or emotional*)) |
| S17 | ("mental health" or "psychological health" or stress or "emotion* regulation*" or "emotional health" or "personal satisfaction" or "quality of life" or "interpersonal relation*" or "social integration" or "social cohesion" or "social adjustment" or "social responsibility" or happiness or "social skill*" or "social identification" or "self-concept" or "self-efficacy" or "sense of coherence" or "social wellbeing" or "social well-being" or "life satisfaction" or "self-esteem" or "self-confidence" or "self-belief*" or "self-advocacy" or "sense of self" or "self-awareness" or "self-acceptance" or "self-control" or "self-regulation" or "self-compassion" or "coping skill*" or "problem solving skill*" or "social connectedness" or "social belonging" or mindfulness) |
| S16 | (MH "Mental Health") OR (MH "Psychological Well-Being") OR (MH "Emotions") OR (MH "Affect") OR (MH "Happiness") OR (MH "Hope") OR (MH "Emotional Regulation") OR (MH "Self Regulation") OR (MH "Personal Satisfaction") OR (MH "Mindfulness") OR (MH "Self-Compassion") OR (MH "Hardiness") OR (MH "Interpersonal Relations") OR (MH "Social Integration") OR (MH "Social Cohesion") OR (MH "Quality of Life") OR (MH "Social Adjustment") OR (MH "Social Skills") OR (MH "Social Responsibility") OR (MH "Social Identity") OR (MH "Self Concept") OR (MH "Self-Efficacy") OR (MH "Self-Awareness") |
| S15 | S9 OR S10 OR S11 OR S12 OR S13 OR S14 |
| S14 | ((child* or boy? or girl?) N3 ("grade 7" or "grade 8" or "grade 9" or "grade 10" or "grade 11" or "grade 12" or "year 7" or "year 8" or "year 9" or "year 10" or "year 11" or "year 12")) |
| S13 | ((child* or boy? or girl?) N2 ("age* twelve" or "age* thirteen" or "age* fourteen" or "age* fifteen" or "age* sixteen" or "age* seventeen" or "age* eighteen" or "age* 12" or "age* 13" or "age* 14" or "age* 15" or "age* 16" or "age* 17" or "age* 18" or "age* of twelve" or "age* of thirteen" or "age* of fourteen" or "age* of fifteen" or "age* of sixteen" or "age* of seventeen" or "age* of eighteen" or "age* of 12" or "age* of 13" or "age* of 14" or "age* of 15" or "age* of 16" or "age* of 17" or "age* of 18")) |
| S12 | ((child* or boy? or girl?) N3 ("12 year? Old?" or "13 year? Old?" or "14 year? Old?" or "15 year? Old?" or "16 year? Old?" or "17 year? Old?" or "18 year? Old?" or "12 years of age" or "13 years of age" or "14 years of age" or "15 years of age" or "16 years of age" or "17 years of age" or "18 years of age")) |
| S11 | ((child* or boy? or girl?) N0 aged N2 ("12 years" or "13 years" or "14 years" or "15 years" or "16 years" or "17 years" or "18 years" or "twelve years" or "thirteen years" or "fourteen years" or "fifteen years" or "sixteen years" or "seventeen years" or "eighteen years")) |
| S10 | (adolescen* or teen* or "secondary school*" or "school student?" or "school girl?" or "school boy?" or "high school" or "middle school" or "grade school") |
| S9 | (MH "Adolescence") OR (MH "Students, High School") OR (MH "Students, Middle School") |
| S8 | S1 OR S2 OR S3 OR S4 OR S5 OR S6 OR S7 |
| S7 | ((embed* or delivered or implemented or integrated or incorporated) N3 (school? or teacher* or highschool*)) |
| S6 | (("tier 1" or "tier one") N0 (intervention* or program*)) and (school or highschool)) |
| S5 | (("system wide" or systemwide or "population wide" or populationwide or "population based" or populationbased) N2 (intervention* or program*)) and (school or highschool)) |
| S4 | ((universal or school or "teacher-led" or teacherled or "student wide" or studentwide or "student based" or studentbased) N2 (intervention* or program* or initiative* or project* or strategy)) |
| S3 | ((classroom or "class room" or schoolbased or "school based" or "school setting*" or "whole school" or "school approach" or "school policies" or "school policy" or "school health polic*" or "school wide" or schoolwide or "school delivered" or "school led" or "teacher led" or "school teacher*" or "school climate" or "school community" or "teaching staff*" or "school staff*" or "core school subject?") N0 (initiative* or project* or strategy)) |
| S2 | ( (curriculum or curricula*) ) OR ( ((classroom or "class room" or schoolbased or "school based" or "school setting*" or "whole school" or "school approach" or "school policies" or "school policy" or "school health polic*" or "school wide" or schoolwide or "school delivered" or "school led" or "teacher led" or "school teacher*" or "school climate" or "school community" or "teaching staff*" or "school staff*" or "core school subject?") N7 (intervention* or program*)) ) |
| S1 | (MH "Curriculum") OR (MH "Schools, Secondary") OR (MH "Schools, Middle") OR (MH "Teachers") OR (MH "School Health Services") |

**Database: EBM Reviews - Cochrane Central Register of Controlled Trials**

| **#** | **Query** |
| --- | --- |
| 1 | curriculum/ or schools/ or School Teachers/ or School Health Services/ |
| 2 | (curriculum or curricula* or ((classroom or class room or schoolbased or school based or school setting* or whole school or school approach or school policies or school policy or school health polic* or school wide or schoolwide or school delivered or school led or teacher led or school teacher* or school climate or school community or teaching staff* or school staff* or core school subject?) adj8 (intervention* or program*))).mp. |
| 3 | ((classroom or class room or schoolbased or school based or school setting* or whole school or school approach or school policies or school policy or school health polic* or school wide or schoolwide or school delivered or school led or teacher led or school teacher* or school climate or school community or school teaching staff* or school staff* or core school subject?) adj (initiative* or project* or strategy)).mp. |
| 4 | ((universal or school or teacher-led or teacherled or student wide or studentwide or student based or studentbased) adj3 (intervention* or program* or initiative* or project* or strategy)).mp. |
| 5 | (((system wide or systemwide or population wide or populationwide or population based or populationbased) adj3 (intervention* or program*)) and (school or highschool)).mp. |
| 6 | (((tier 1 or tier one) adj (intervention* or program*)) and (school or highschool)).mp. |
| 7 | ((embed* or delivered or implemented or integrated or incorporated) adj4 (school? or teacher* or highschool*)).mp. |
| 8 | 1 or 2 or 3 or 4 or 5 or 6 or 7 |
| 9 | Adolescent/ or Students/ |
| 10 | (adolescen* or teen* or secondary school* or school student? or school girl? or school boy? or high school or middle school or grade school).mp. |
| 11 | ((child* or boy? or girl?) adj aged adj3 (12 years or 13 years or 14 years or 15 years or 16 years or 17 years or 18 years or twelve years or thirteen years or fourteen years or fifteen years or sixteen years or seventeen years or eighteen years)).mp. |
| 12 | ((child* or boy? or girl?) adj4 (12 year? Old? or 13 year? Old? or 14 year? Old? or 15 year? Old? or 16 year? Old? or 17 year? Old? or 18 year? Old? or 12 years of age or 13 years of age or 14 years of age or 15 years of age or 16 years of age or 17 years of age or 18 years of age)).mp. |
| 13 | ((child* or boy? or girl?) adj3 (age* twelve or age* thirteen or age* fourteen or age* fifteen or age* sixteen or age* seventeen or age* eighteen or age* 12 or age* 13 or age* 14 or age* 15 or age* 16 or age* 17 or age* 18 or age* of twelve or age* of thirteen or age* of fourteen or age* of fifteen or age* of sixteen or age* of seventeen or age* of eighteen or age* of 12 or age* of 13 or age* of 14 or age* of 15 or age* of 16 or age* of 17 or age* of 18)).mp. |
| 14 | ((child* or boy? or girl?) adj4 (grade 7 or grade 8 or grade 9 or grade 10 or grade 11 or grade 12 or year 7 or year 8 or year 9 or year 10 or year 11 or year 12)).mp. |
| 15 | 9 or 10 or 11 or 12 or 13 or 14 |
| 16 | Mental health/ or Emotions/ or Psychological wellbeing/ or Personal satisfaction/ or mindfulness/ or resilience, psychological/ or happiness/ or hope/ or Emotional regulation/ or affect/ or interpersonal relations/ or quality of life/ or Social integration/ or Social cohesion/ or Social adjustment/ or Social responsibility/ or Happiness/ or Social skills/ or Social identification/ or Self-concept/ or Self-efficacy/ or Sense of coherence/ |
| 17 | (mental health or psychological health or stress or emotion* regulation* or emotional health or personal satisfaction or quality of life or interpersonal relation* or social integration or social cohesion or social adjustment or social responsibility or happiness or social skill* or social identification or self-concept or self-efficacy or sense of coherence or social wellbeing or social well-being or life satisfaction or self-esteem or self-confidence or self-belief* or self-advocacy or sense of self or self-awareness or self-acceptance or self-control or self-regulation or self-compassion or coping skill* or problem solving skill* or social connectedness or social belonging or mindfulness).mp. |
| 18 | ((wellness or wellbeing or well-being or empowerment or resilience or resilient or flourishing or thriving or coping) adj5 (psychological* or mental* or emotional*)).mp. |
| 19 | mental disorders/ or anxiety disorders/ or obsessive-compulsive disorder/ or panic disorder/ or phobic disorders/ or phobia, social/ or "disruptive, impulse control, and conduct disorders"/ or dissociative disorders/ or dissociative identity disorder/ or "feeding and eating disorders"/ or anorexia nervosa/ or avoidant restrictive food intake disorder/ or binge-eating disorder/ or bulimia nervosa/ or diabulimia/ or "feeding and eating disorders of childhood"/ or mood disorders/ or "bipolar and related disorders"/ or depressive disorder/ or cyclothymic disorder/ or attention deficit disorder with hyperactivity/ or conduct disorder/ or child behavior disorders/ or reactive attachment disorder/ or schizophrenia, childhood/ or personality disorders/ or antisocial personality disorder/ or borderline personality disorder/ or compulsive personality disorder/ or dependent personality disorder/ or histrionic personality disorder/ or paranoid personality disorder/ or passive-aggressive personality disorder/ or schizoid personality disorder/ or schizotypal personality disorder/ or "schizophrenia spectrum and other psychotic disorders"/ or affective disorders, psychotic/ or psychotic disorders/ or psychoses, substance-induced/ or schizophrenia/ or schizophrenia, catatonic/ or schizophrenia, disorganized/ or schizophrenia, paranoid/ or schizophrenia, treatment-resistant/ or somatoform disorders/ or body dysmorphic disorders/ or body integrity identity disorder/ or conversion disorder/ or factitious disorders/ or munchausen syndrome/ or munchausen syndrome by proxy/ or substance-related disorders/ or alcohol-related disorders/ or alcoholic intoxication/ or alcoholism/ or binge drinking/ or psychoses, alcoholic/ or amphetamine-related disorders/ or cocaine-related disorders/ or inhalant abuse/ or marijuana abuse/ or "marijuana use"/ or narcotic-related disorders/ or neonatal abstinence syndrome/ or phencyclidine abuse/ or substance abuse, intravenous/ or substance abuse, oral/ or "trauma and stressor related disorders"/ or adjustment disorders/ or stress disorders, traumatic/ or stress disorders, post-traumatic/ or stress, psychological/ or burnout, psychological/ or adolescent behavior/ or underage drinking/ or behavioral symptoms/ or affective symptoms/ or delusions/ or depersonalization/ or depression/ or obsessive behavior/ or paranoid behavior/ or problem behavior/ or self-injurious behavior/ or self mutilation/ or suicide/ or suicidal ideation/ or suicide, attempted/ or suicide, completed/ or impulsive behavior/ or compulsive behavior/ or "marijuana use"/ or marijuana smoking/ or "recreational drug use"/ or risk-taking/ or bullying/ or cyberbullying/ |
| 20 | ((mental or anxiety or depression or neurotic or obsessive-compulsive or compulsive-obsessional or panic or phobic or bipolar or disruptive or impulse control or conduct or dissociative or identity or eating or mood or depressive or dysthymic or affective or attention deficit or behavio* or hyperactivity or autism spectrum or autistic or paraphilic or personality or psychotic or paranoid or somatoform or somati#ation or somatic symptom or psychophysiologic* or psychosomatic or body dysmorphic or body integrity identity or stressor related or adjustment or traumatic stress or post-traumatic or posttraumatic or acute traumatic or emotional or disruptive mood dysregulation or substance induced or medication induced or alcohol induced or substance related or alcohol related or drug induced or hoarding or attachment or conversion or bodily distress or functional movement or functional neurological or functional neurologic symptom or sleep wake or sleep initiation or sleep maintenance or insomnia or oppositional defiant or intermittent explosive or factitious or psychoneurotic or manic or cyclothymic or autophag* or schizoaffective or unipolar or delusional or addiction or abnormal psychology) adj disorder*).mp. |
| 21 | (mental illness or psychological illness or psychological distress or mental distress or psychological stress or self-harm or risk taking or risk behavio?r* or suicide or suicidal or bullying or cyberbullying or aggressive behavio?r* or disruptive behavio?r* or behavio?ral problem*).mp. |
| 22 | ((drug* or substanc* or narcotic* or chemical* or heroin* or methamphetamin* or amphetamin* or cocain* or crack* or ice* or tobacco* or smok* or cigarett* or nicotin* or alcohol* or "crystal meth*" or speed* or benzodiazepin* or cannabis* or marijuana*) adj (abuse* or addict* or misuse* or dependen*)).mp. |
| 23 | 16 or 17 or 18 or 19 or 20 or 21 or 22 |
| 24 | 8 and 15 and 23 |
| 25 | 24 not (case report* or comment* or editorial or letter or news*).ti. |
| 26 | 24 not review*.ti. |

**Database: SCOPUS**

(((TITLE-ABS-KEY(( (curriculum or curricula*) ) OR ( ((classroom or "class room" or schoolbased or "school based" or "school setting*" or "whole school" or "school approach" or "school policies" or "school policy" or "school health polic*" or "school wide" or schoolwide or "school delivered" or "school led" or "teacher led" or "school teacher*" or "school climate" or "school community" or "teaching staff*" or "school staff*" or "core school subject?") W/7 (intervention* or program*)) ))) OR (TITLE-ABS-KEY(((classroom or "class room" or schoolbased or "school based" or "school setting*" or "whole school" or "school approach" or "school policies" or "school policy" or "school health polic*" or "school wide" or schoolwide or "school delivered" or "school led" or "teacher led" or "school teacher*" or "school climate" or "school community" or "teaching staff*" or "school staff*" or "core school subject?") W/0 (initiative* or project* or strategy)))) OR (TITLE-ABS-KEY(((universal or school or "teacher-led" or teacherled or "student wide" or studentwide or "student based" or studentbased) W/2 (intervention* or program* or initiative* or project* or strategy)))) OR (TITLE-ABS-KEY(( ( "system wide" OR systemwide OR "population wide" OR populationwide OR "population based" OR populationbased ) W/2 ( intervention* OR program* ) ) AND ( school OR highschool))) OR (TITLE-ABS-KEY(((embed* or delivered or implemented or integrated or incorporated) W/3 (school? or teacher* or highschool*))))) AND (((TITLE-ABS-KEY((adolescen* or teen* or "secondary school*" or "school student?" or "school girl?" or "school boy?" or "high school" or "middle school" or "grade school")) OR TITLE-ABS-KEY(((child* or boy? or girl?) W/0 aged W/2 ("12 years" or "13 years" or "14 years" or "15 years" or "16 years" or "17 years" or "18 years" or "twelve years" or "thirteen years" or "fourteen years" or "fifteen years" or "sixteen years" or "seventeen years" or "eighteen years"))))) OR ((TITLE-ABS-KEY(((child* or boy? or girl?) W/3 ("12 year? Old?" or "13 year? Old?" or "14 year? Old?" or "15 year? Old?" or "16 year? Old?" or "17 year? Old?" or "18 year? Old?" or "12 years of age" or "13 years of age" or "14 years of age" or "15 years of age" or "16 years of age" or "17 years of age" or "18 years of age"))) OR TITLE-ABS-KEY(((child* or boy? or girl?) W/2 ("age* twelve" or "age* thirteen" or "age* fourteen" or "age* fifteen" or "age* sixteen" or "age* seventeen" or "age* eighteen" or "age* 12" or "age* 13" or "age* 14" or "age* 15" or "age* 16" or "age* 17" or "age* 18" or "age* of twelve" or "age* of thirteen" or "age* of fourteen" or "age* of fifteen" or "age* of sixteen" or "age* of seventeen" or "age* of eighteen" or "age* of 12" or "age* of 13" or "age* of 14" or "age* of 15" or "age* of 16" or "age* of 17" or "age* of 18"))) OR TITLE-ABS-KEY(((child* or boy? or girl?) W/3 ("grade 7" or "grade 8" or "grade 9" or "grade 10" or "grade 11" or "grade 12" or "year 7" or "year 8" or "year 9" or "year 10" or "year 11" or "year 12")))))) AND (((TITLE-ABS-KEY(("mental health" or "psychological health" or stress or "emotion* regulation*" or "emotional health" or "personal satisfaction" or "quality of life" or "interpersonal relation*" or "social integration" or "social cohesion" or "social adjustment" or "social responsibility" or happiness or "social skill*" or "social identification" or "self-concept" or "self-efficacy" or "sense of coherence" or "social wellbeing" or "social well-being" or "life satisfaction" or "self-esteem" or "self-confidence" or "self-belief*" or "self-advocacy" or "sense of self" or "self-awareness" or "self-acceptance" or "self-control" or "self-regulation" or "self-compassion" or "coping skill*" or "problem solving skill*" or "social connectedness" or "social belonging" or mindfulness)) OR TITLE-ABS-KEY(((wellness or wellbeing or "well-being" or empowerment or resilience or resilient or flourishing or thriving or coping) W/4 (psychological* or mental* or emotional*))) OR TITLE-ABS-KEY(((mental or anxiety or depression or neurotic or "obsessive-compulsive" or "compulsive-obsessional" or panic or phobic or bipolar or disruptive or "impulse control" or conduct or dissociative or identity or eating or mood or depressive or dysthymic or affective or "attention deficit" or behavio* or hyperactivity or "autism spectrum" or autistic or paraphilic or personality or psychotic or paranoid or somatoform or somati#ation or "somatic symptom" or psychophysiologic* or psychosomatic or "body dysmorphic" or "body integrity identity" or "stressor related" or adjustment or "traumatic stress" or "post-traumatic" or posttraumatic or "acute traumatic" or emotional or "disruptive mood dysregulation" or "substance induced" or "medication induced" or "alcohol induced" or "substance related" or "alcohol related" or "drug induced" or hoarding or attachment or conversion or "bodily distress" or "functional movement" or "functional neurological" or "functional neurologic symptom" or "sleep wake" or "sleep initiation" or "sleep maintenance" or insomnia or "oppositional defiant" or "intermittent explosive" or factitious or psychoneurotic or manic or cyclothymic or autophag* or schizoaffective or unipolar or delusional or addiction or "abnormal psychology") W/0 disorder*)))) OR ((TITLE-ABS-KEY(("mental illness" or "psychological illness" or "psychological distress" or "mental distress" or "psychological stress" or "self-harm" or "risk taking" or "risk behavio?r*" or suicide or suicidal or bullying or cyberbullying or "aggressive behavio?r*" or "disruptive behavio?r*" or "behavio?ral problem*")) OR TITLE-ABS-KEY(((drug* or substanc* or narcotic* or chemical* or heroin* or methamphetamin* or amphetamin* or cocain* or crack* or ice* or tobacco* or smok* or cigarett* or nicotin* or alcohol* or "crystal meth*" or speed* or benzodiazepin* or cannabis* or marijuana*) W/0 (abuse* or addict* or misuse* or dependen*)))))) AND ((TITLE-ABS-KEY("clinical trial*" or "Random Assignment" or "randomi* control* trial*" or "random* allocat*" or "allocat* random*" or "allocat* at random" or quasirandom* or "Quantitative Stud*") OR TITLE-ABS-KEY((singl* W/1 blind*) or (singl* W/1 mask*) or (doubl* W/1 blind*) or (doubl* W/1 mask*) or (tripl* W/1 blind*) or (tripl* W/1 mask*) or (trebl* W/1 blind*) or (trebl* W/1 mask*)) OR TITLE-ABS-KEY(placebo) OR TITLE((trial or trials))))) AND NOT (TITLE((Anecdote OR "Book Review" OR "Brief Item" OR Commentary OR Dissertation OR Editorial OR "Legal Case" OR Letter OR "Masters Thesis" OR Pamphlet OR Review* OR "Systematic Review" OR Website))) AND ( EXCLUDE ( DOCTYPE,"re" ) OR EXCLUDE ( DOCTYPE,"ed" ) OR EXCLUDE ( DOCTYPE,"no" ) OR EXCLUDE ( DOCTYPE,"er" ) OR EXCLUDE ( DOCTYPE,"sh" ) ) AND ( LIMIT-TO ( LANGUAGE,"English" ) )

**Database: ERIC**

noft(curriculum or curricula* )

noft(((classroom or "class room" or schoolbased or "school based" or "school setting*" or "whole school" or "school approach" or "school policies" or "school policy" or "school health polic*" or "school wide" or schoolwide or "school delivered" or "school led" or "teacher led" or "school teacher*" or "school climate" or "school community" or "teaching staff*" or "school staff*" or "core school subject?") N/7 (intervention* or program*)))

noft(((classroom OR "class room" OR schoolbased OR "school based" OR "school setting*" OR "whole school" OR "school approach" OR "school policies" OR "school policy" OR "school health polic*" OR "school wide" OR schoolwide OR "school delivered" OR "school led" OR "teacher led" OR "school teacher*" OR "school climate" OR "school community" OR "teaching staff*" OR "school staff*" OR "core school subject?") N/0 (initiative* or project* or strategy)))

noft(((universal or school or "teacher-led" or teacherled or "student wide" or studentwide or "student based" or studentbased) N/2 (intervention* or program* or initiative* or project* or strategy)))

noft((("system wide" or systemwide or "population wide" or populationwide or "population based" or populationbased) N/2 (intervention* or program*))) AND noft((school or highschool))

noft((("tier 1" or "tier one") N/0 (intervention* or program*)) ) AND noft(school or highschool)

noft(((embed* or delivered or implemented or integrated or incorporated) N/3 (school? or teacher* or highschool*)))

noft([S1]) OR noft([S2]) OR noft([S3]) OR noft([S4]) OR noft([S5]) OR noft([S6]) OR noft([S7])

noft(("mental health" or "psychological health" or stress or "emotion* regulation*" or "emotional health" or "personal satisfaction" or "quality of life" or "interpersonal relation*" or "social integration" or "social cohesion" or "social adjustment" or "social responsibility" or happiness or "social skill*" or "social identification" or "self-concept" or "self-efficacy" or "sense of coherence" or "social wellbeing" or "social well-being" or "life satisfaction" or "self-esteem" or "self-confidence" or "self-belief*" or "self-advocacy" or "sense of self" or "self-awareness" or "self-acceptance" or "self-control" or "self-regulation" or "self-compassion" or "coping skill*" or "problem solving skill*" or "social connectedness" or "social belonging" or mindfulness))

noft(((wellness or wellbeing or "well-being" or empowerment or resilience or resilient or flourishing or thriving or coping) N/4 (psychological* or mental* or emotional*)))

noft(((mental or anxiety or depression or neurotic or “obsessive-compulsive” or “compulsive-obsessional” or panic or phobic or bipolar or disruptive or “impulse control” or conduct or dissociative or identity or eating or mood or depressive or dysthymic or affective or “attention deficit” or behavio* or hyperactivity or “autism spectrum” or autistic or paraphilic or personality or psychotic or paranoid or somatoform or somati#ation or “somatic symptom” or psychophysiologic* or psychosomatic or “body dysmorphic” or “body integrity identity” or “stressor related” or adjustment or “traumatic stress” or “post-traumatic” or posttraumatic or “acute traumatic” or emotional or “disruptive mood dysregulation” or “substance induced” or “medication induced” or “alcohol induced” or “substance related” or “alcohol related” or “drug induced” or hoarding or attachment or conversion or “bodily distress” or “functional movement” or “functional neurological” or “functional neurologic symptom” or “sleep wake” or “sleep initiation” or “sleep maintenance” or insomnia or “oppositional defiant” or “intermittent explosive” or factitious or psychoneurotic or manic or cyclothymic or autophag* or schizoaffective or unipolar or delusional or addiction or “abnormal psychology”) N/0 disorder*))

noft((“mental illness” or “psychological illness” or “psychological distress” or “mental distress” or “psychological stress” or “self-harm” or “risk taking” or “risk behavio?r*” or suicide or suicidal or bullying or cyberbullying or “aggressive behavio?r*” or “disruptive behavio?r*” or “behavio?ral problem*”))

noft(((drug* or substanc* or narcotic* or chemical* or heroin* or methamphetamin* or amphetamin* or cocain* or crack* or ice* or tobacco* or smok* or cigarett* or nicotin* or alcohol* or "crystal meth*" or speed* or benzodiazepin* or cannabis* or marijuana*) N/0 (abuse* or addict* or misuse* or dependen*)))

noft([S15]) OR noft([S16]) OR noft([S17]) OR noft([S18]) OR noft([S19])

"clinical trial*" or "Random Assignment" or "randomi* control* trial*" or "random* allocat*" or "allocat* random*" or "allocat* at random" or quasirandom* or "Quantitative Stud*"

(singl* N/1 blind*) or (singl* N/1 mask*) or (doubl* N/1 blind*) or (doubl* N/1 mask*) or (tripl* N/1 blind*) or (tripl* N/1 mask*) or (trebl* N/1 blind*) or (trebl* N/1 mask*)

noft(placebo*) AND title(trial or trials)

noft([S21]) OR noft([S22]) OR noft([S23])
